# Two-dimensional molecular networks at the solid/liquid interface and the role of alkyl chains in their building blocks

**DOI:** 10.3762/bjnano.14.72

**Published:** 2023-08-23

**Authors:** Suyi Liu, Yasuo Norikane, Yoshihiro Kikkawa

**Affiliations:** 1 Graduate School of Science and Technology, University of Tsukuba, Ibaraki, 305-8571, Japan; 2 National Institute of Advanced Industrial Science and Technology (AIST), Tsukuba Central 5, 1-1-1 Higashi, Tsukuba, Ibaraki 305-8565, Japan; 3 Faculty of Pure and Applied Sciences, University of Tsukuba, Ibaraki, 305-8571, Japan

**Keywords:** alkyl chains, scanning tunneling microscopy, self-assembly, solid/liquid interface, two-dimensional networks

## Abstract

Nanoarchitectonics has attracted increasing attention owing to its potential applications in nanomachines, nanoelectronics, catalysis, and nanopatterning, which can contribute to overcoming global problems related to energy and environment, among others. However, the fabrication of ordered nanoarchitectures remains a challenge, even in two dimensions. Therefore, a deeper understanding of the self-assembly processes and substantial factors for building ordered structures is critical for tailoring flexible and desirable nanoarchitectures. Scanning tunneling microscopy is a powerful tool for revealing the molecular conformations, arrangements, and orientations of two-dimensional (2D) networks on surfaces. The fabrication of 2D assemblies involves non-covalent interactions that play a significant role in the molecular arrangement and orientation. Among the non-covalent interactions, dispersion interactions that derive from alkyl chain units are believed to be weak. However, alkyl chains play an important role in the adsorption onto substrates, as well as in the in-plane intermolecular interactions. In this review, we focus on the role of alkyl chains in the formation of ordered 2D assemblies at the solid/liquid interface. The alkyl chain effects on the 2D assemblies are introduced together with examples documented in the past decades.

## Introduction

The fabrication of ordered nanostructures using the concept of nanoarchitectonics [1–4] for various applications such as nanomachines, nanoelectronics, catalysis, and nanopatterning remains challenging [5–7]. Design and synthesis of molecular building blocks have enabled the construction of well-organized nanoarchitectures with various dimensions [8–11]. These characteristic structural formations are governed by self-assembly processes via non-covalent intermolecular interactions, such as hydrogen bonding, metal coordination, halogen bonding, and dispersion forces [12–22]. Scanning tunneling microscopy (STM) is an important tool for the direct visualization of molecular arrangements, especially for two-dimensional (2D) networks. STM observations have been performed on atomically flat conducting substrates, such as metal surfaces and highly oriented pyrolytic graphite (HOPG), under ultrahigh vacuum (UHV) conditions, at solid/air or solid/liquid interfaces [23–28]. Although UHV-STM offers high-resolution imaging, it requires large, complex, and expensive instruments as well as thermally stable samples that do not decompose under sublimation during sample preparation. By contrast, STM at the solid/liquid interface is efficient for various sample types and requires only a simple apparatus [24].

**Figure 1:**
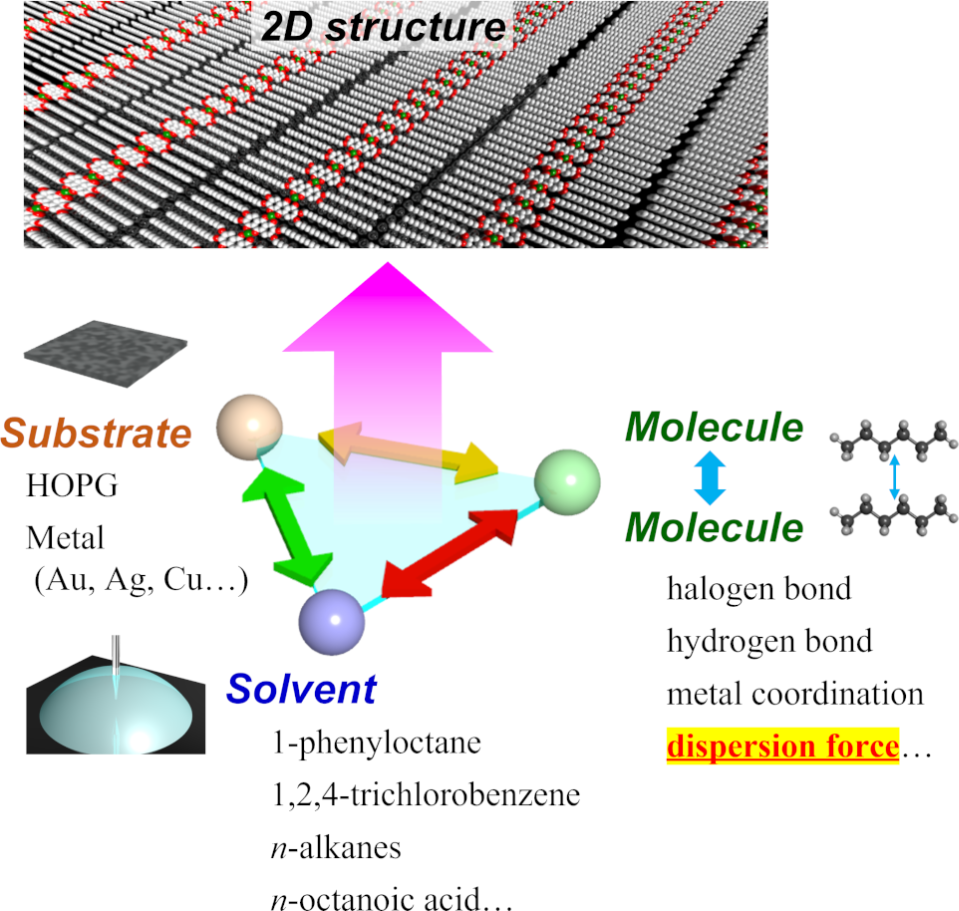
Intermolecular interactions related to the formation of 2D assemblies at the solid/liquid interface.

Physisorbed monolayers at the solid/liquid interface have been extensively studied for revealing the supramolecular interactions in the formation of self-assembled monolayers [29–34]. Several intermolecular interactions take place at the solid/liquid interface that should be taken into account for the controlled molecular organization in two dimensions, namely molecule–molecule, molecule–substrate, and solvent–molecule interactions (Figure 1). The target molecules are dissolved in non-conductive solvents with low volatility, such as 1-phenyloctane, 1,2,4-trichlorobenzene (TCB), long-chain *n*-alkanes, and octanoic acid [35–37]. The physisorbed monolayers can be prepared by simply placing the sample solution on a freshly cleaved HOPG surface. The self-assembly at the solid/liquid interface is characterized by the following properties: (i) The correlation between the molecular structure and resultant 2D arrangements can be revealed by STM with high resolution. (ii) Post-reaction is available by external stimuli such as the addition of metal ions for metal coordination, light irradiation for photoreaction, and post-blending of other molecules. (iii) The dynamic process of the 2D structural change (caused by the external stimuli) can be followed in situ and in real time [38–42].

In the 2D assemblies at the solid/liquid interface, non-covalent interactions play an essential role in the molecular orientation and arrangement. Among the intermolecular interactions, directional and relatively strong interactions, such as hydrogen bonding, halogen bonding, and metal coordination, are often exploited for the formation of 2D structures. However, dispersion forces originating from the alkyl chains also play an important role in the adsorption onto the substrate, as well as in the in-plane intermolecular interactions at the solid/liquid interface, apart from being the solubilizing agent.

A comprehensive review related to the odd–even effect was reported in 2007 [43], but other effects of alkyl chains also play important roles in the formation of 2D molecular networks and have not yet been summarized in a review. In this review, we focus on the effects of alkyl chains on 2D structure formation at the HOPG/solvent interface and we report some examples of the past decades. We present the effect of the alkyl chain on 2D structure formation either alone or combined with other non-covalent interactions. We then discuss the essential role of alkyl chains in 2D nanoarchitectures at the solid/liquid interface.

## Review

### 1 Adsorption of alkyl chains

**Figure 2:**
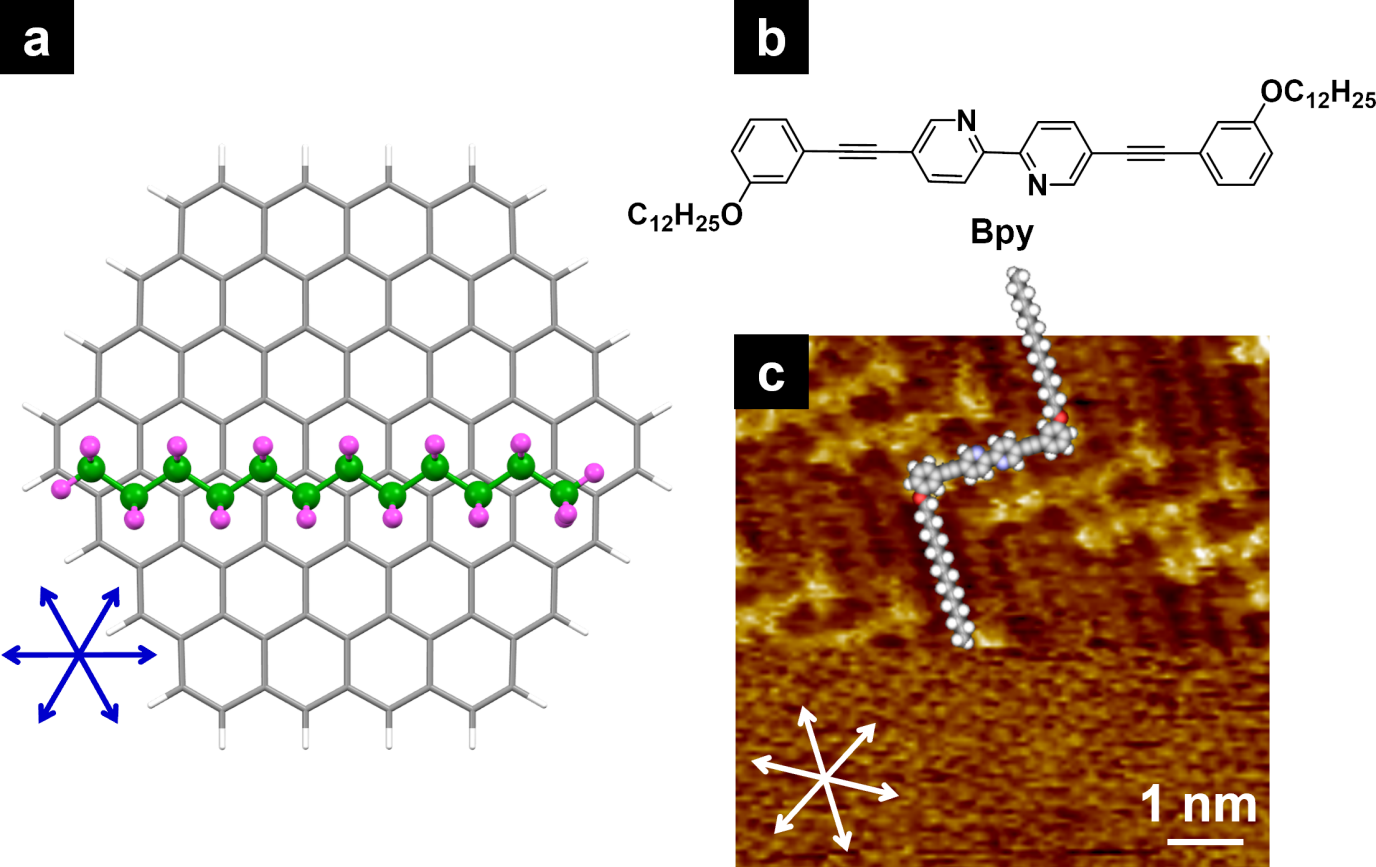
(a) DFT-optimized geometry of *n*-dodecane adsorbed on C_96_H_24_ as a HOPG model. The carbon and hydrogen atoms of adsorbed *n*-dodecane are colored in green and pink, respectively. Geometry optimization was performed using the Gaussian 16 program [50] at the B3LYP/6-31G* level [51] with Grimme’s D3 dispersion correction [52]. The optimized geometry of isolated C_96_H_24_ was fixed during the calculation, and the geometry of the adsorbed *n*-dodecane was optimized. Similar DFT calculations are reported in [47]. The blue arrows indicate the lattice directions of C_96_H_24_. (b) Chemical structure of a bipyridine derivative (**Bpy**), and (c) STM image of the **Bpy** monolayer at the HOPG/1-phenyloctane interface. Both molecular (upper) and HOPG lattice images (lower) were obtained by altering the tunneling conditions: (upper) *I* = 1 pA, *V* = −1000 mV; (lower) *I* = 500 pA, *V* = 30 mV. The white arrows indicate the threefold symmetric axes of the HOPG lattice.

The first step in the formation of self-assembled structures at the HOPG/solvent interface is the adsorption of molecules via dispersion forces deriving from alkyl chains and other moieties such as aromatic units. Generally, dispersion forces are interpreted as non-directional interactions. However, the interaction between HOPG and alkyl chains causes directional orientation because of the epitaxy defined by the threefold symmetric axis of the HOPG lattice, that is, alkyl chains align along the HOPG lattice directions [44]. Orientation of alkyl chain backbone on the HOPG surface has also been discussed. There are edge-on and flat-on orientations, in which the all-trans zigzag plane of the alkyl chain is perpendicular and parallel to the surface, respectively. Edge-on oriented alkyl chain adsorption on HOPG has been reported in some cases [44–46], but the flat-on orientation is more favorable [47–49]. For an example of the flat-on orientation, density functional theory (DFT) calculations revealed the optimized geometry of *n*-dodecane adsorbed onto C_96_H_24_ as a HOPG model (Figure 2a). The hydrogen atoms of *n*-dodecane with a trans zigzag conformation are located near the centers of the six-membered rings of C_96_H_24_, and the molecule is oriented along one of the lattice directions of C_96_H_24_, indicated by the blue arrows. In STM imaging, changes in bias voltage (*V*) and tunneling current (*I*) enable the capture of both molecular arrangements and the HOPG lattice images. As shown in Figure 2b and Figure 2c, the alkyl chains of a bipyridine derivative follow one of the HOPG lattice directions, as indicated by the white arrows. Image corrections using the HOPG lattice (periodicity of 0.246 nm) as a reference can provide precise 2D structures, including intermolecular distances and molecular orientations.

To study the adsorption of alkane on graphite, computational simulations such as molecular mechanics and DFT calculations with the local density approximation have been applied [48–49,53–55]. Recently, dispersion-corrected DFT calculations have quantitatively revealed the interactions between *n*-alkanes and circumcoronene as models of molecular adsorption on HOPG [47]. As the number of carbon atoms in the *n*-alkane increased, the adsorption energy increased by −1.85 kcal/mol per CH_2_ unit. The absolute value is almost identical to the desorption energy obtained by temperature-programmed desorption measurements (1.90 kcal/mol per CH_2_ unit) [56]. This result suggests that the longer the alkyl chain, the larger the proportion of stabilization energy caused by the alkyl chains in the entire system. Furthermore, the alkyl chains exhibit lateral interactions upon dense packing, and the dispersion interactions increased by −0.50 kcal/mol per CH_2_ unit [47]. Although alkyl chains basically follow the HOPG lattice, lattice mismatch between *n*-alkanes and graphite has been reported for very long alkyl chains [57]. The combination of dispersion-corrected DFT calculations and STM visualizations revealed that a swerved chain conformation of *n*-alkanes appeared when the chain length reached a critical length (typically over C50). Note that even shorter alkyl chains with functionalized group have been reported to exhibit distorted adsorption on HOPG in some cases [44,58–61].

This review mainly focuses on the alkyl chain effects on the HOPG surface. However, it is important to note that the kinds of substrate have influence on the 2D molecular self-assemblies. 2D structures on metallic surfaces, such as Au(111), are different from those on a HOPG surface, even if the molecular building blocks are the same [62–64]. This is because of the different molecule–substrate interactions on Au(111) and HOPG. The adsorption energy of alkyl chains on Au(111) has been reported as −1.48 kcal/mol per CH_2_ unit [65], whereas that on HOPG is approximately −1.9 kcal/mol per CH_2_ unit [47,56], as noted above. The periodicity of alkyl chains almost matches HOPG lattice, but does not Au(111) lattice, on which the alkyl chains favor to align along the nearest neighbor direction [66]. These differences in the dispersion interaction may be one of the causes of 2D structural changes between Au(111) and HOPG surfaces [67]. In the following sections, the effect of alkyl chains on 2D structure formations are summarized only for the HOPG surface.

### 2 Missing alkyl chains

Although the alkyl chains contribute to the adsorption onto HOPG, in some cases, the number of adsorbed alkyl chains in 2D molecular networks is small compared to the number of alkyl chains originally present in the adsorbate molecule. This phenomenon has been explained by the dangling of alkyl chains toward the solvent phase or the double-deck assembly.

**Scheme 1:**
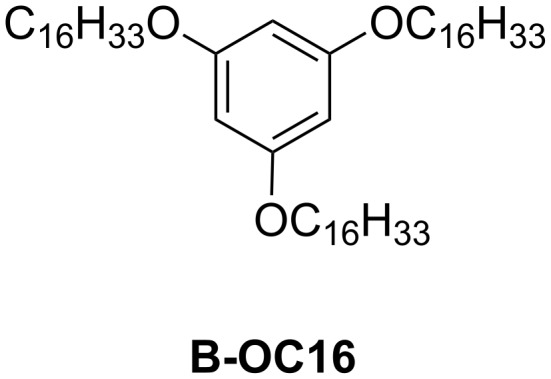
Chemical structure of **B-OC16** [71].

**Figure 3:**
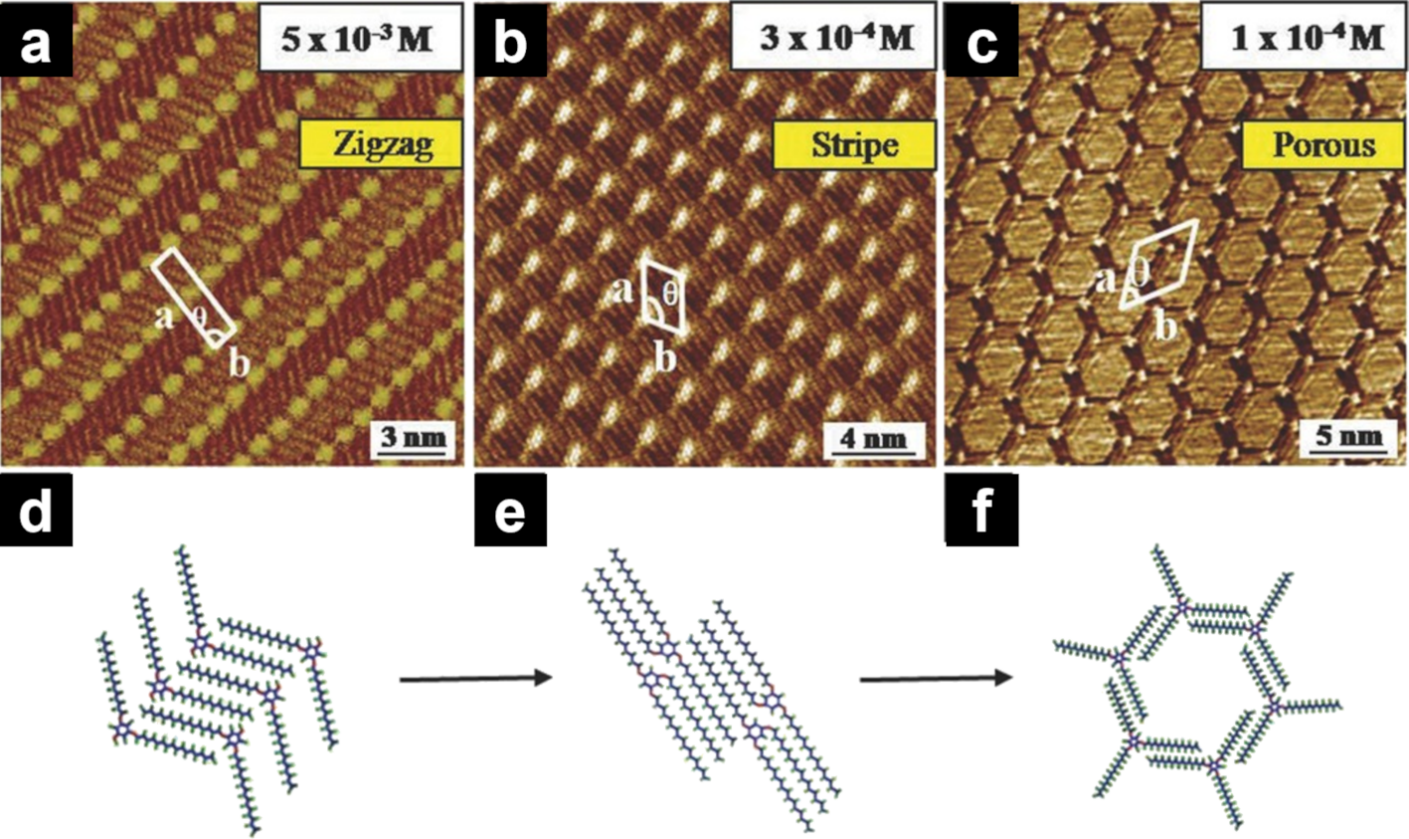
(a–c) STM images of the **B-OC16** physisorbed monolayers at the HOPG/1-phenyloctane interface and (d–f) corresponding molecular models. (a, d) zigzag structure, (b, e) striped pattern, and (c, f) porous structure. The number of adsorbed alkyl chains varies within the 2D structures and depends on the concentration. Figure 3 was adapted from [71], X. Shen et al. “Concentration-Controlled Reversible Phase Transitions in Self-Assembled Monolayers on HOPG Surfaces", Small, with permission from John Wiley and Sons. Copyright © 2015 WILEY-VCH Verlag GmbH & Co. KGaA, Weinheim. This content is not subject to CC BY 4.0.

Since non-adsorbed alkyl chains cannot be detected by STM, the missing (non-observed) alkyl chains are considered as dangling into the solvent phase [68–70]. For example, physisorbed monolayers of 1,3,5-tetra(hexadecyloxy)benzene (**B-OC16**: Scheme 1) were prepared at the HOPG/1-phenyloctane interface [71]. STM observations revealed the concentration dependence of the formation of 2D structures. At the highest concentration (5.0 × 10^−3^ M), two of the three alkyl chains attached to HOPG interacted via dispersion forces to form a zigzag structure (Figure 3a,d). One of the three alkyl chains was considered to deviate from the HOPG surface. Upon decreasing the concentration of the solution either in situ (adding more solvent to HOPG) or ex situ (simple dilution), all alkyl chains adsorbed on the HOPG surface formed striped (Figure 3b,e) or porous structures (Figure 3c,f). Thus, the adsorption of alkyl chains in the building blocks is sometimes affected by the concentration of the sample.

**Scheme 2:**
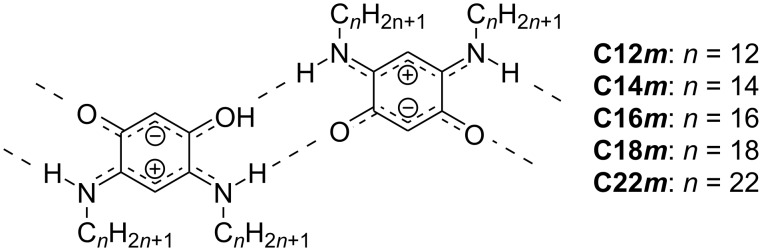
Chemical structure of *m*-quinonemonoimines with different alkyl chain lengths (**C12*m***–**C22*m***) [72].

**Figure 4:**
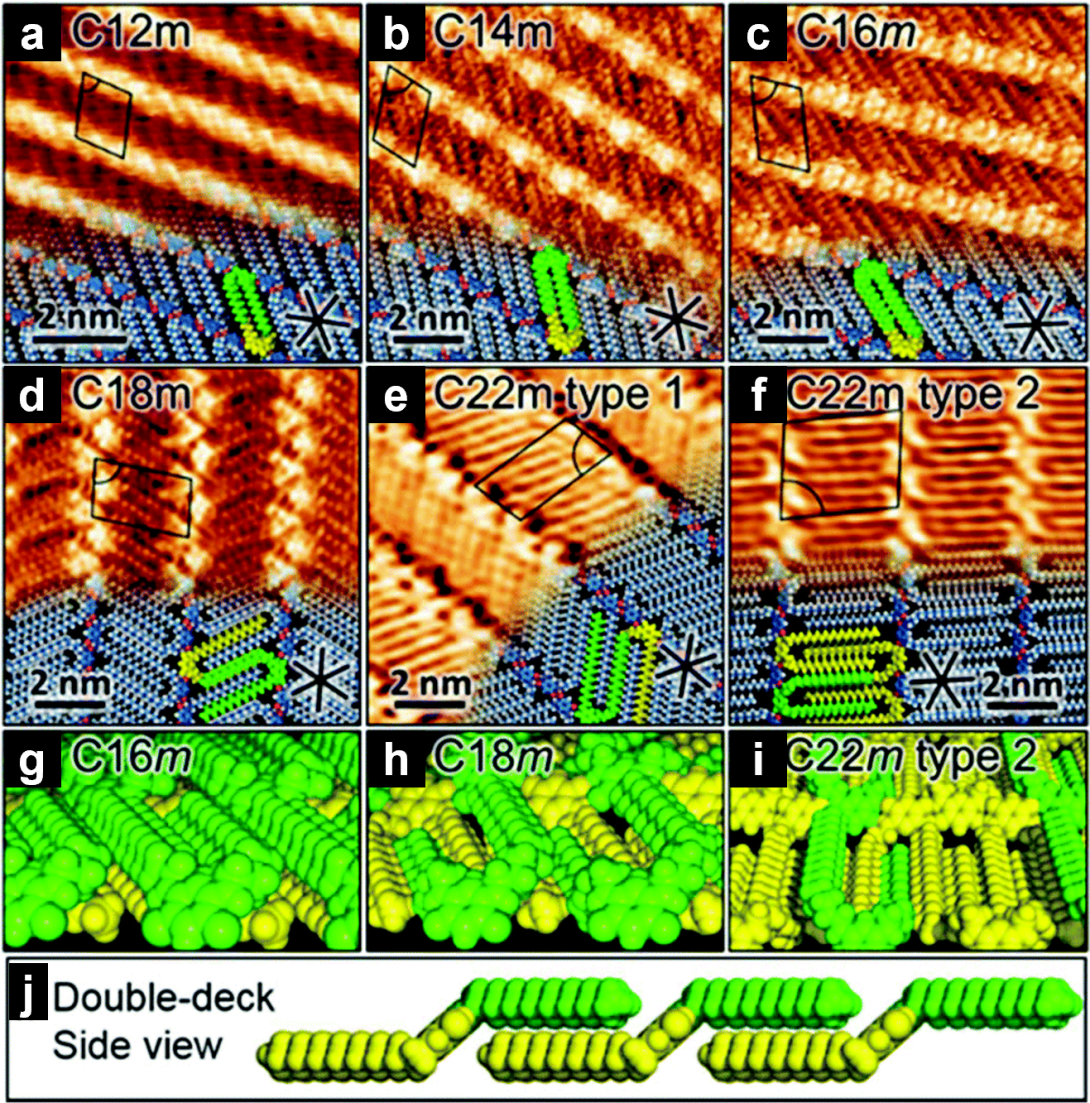
(a–f) STM images formed at the HOPG/TCB interface. (a–c) **C12*m***, **C14*m***, and **C16*m*** form complete double-deck assemblies. The molecules highlighted in green and yellow overlap. (d) **C18*m*** forms a partial double-deck packing. (e, f) **C22*m*** forms two types of domains: one is composed of partial double-deck packing (e), and the other one is constructed by the mixture of partial double-deck packing and completely adsorbed molecules (f). (g–i) Tilted view of the double-deck assembly. (j) Side view of the complete double-deck packing (**C16*m***). The black lines indicate the threefold symmetric axes of the HOPG lattice. Figure 4 was adapted with permission of The Royal Society of Chemistry, from [72], (“Alkyl chain length effects on double-deck assembly at a liquid/solid interface” by Y. Fang et al., Nanoscale, Vol. 10, Issue 31, © 2018); permission conveyed through Copyright Clearance Center, Inc. This content is not subject to CC BY 4.0.

Regarding double-deck assemblies, 2D assemblies of zwitterionic *m*-quinonemonoimines with different alkyl chain lengths (Scheme 2) were studied using STM at the HOPG/TCB interface [72]. In Figure 4a–c, the alkyl chains of **C12*m***, **C14*m***, and **C16*m*** formed a complete double-deck assembly (all alkyl chains overlapped, as shown in the tilted and side views of Figure 4g and Figure 4j, respectively), whereas those of C18 contained a partial double deck (one of the alkyl chains overlapped, Figure 4d,h). In the case of C22, both partial double-deck assembly and completely adsorbed alkyl chains were observed in different domains owing to the increased adsorption energy afforded by the chain length (Figure 4f,i). The balance between alkyl chain adsorption on a surface and conformational distortion near the head group determines the type of double-deck assembly.

### 3 Chain length

#### 3.1 Intercolumnar distances and structural changes

**Scheme 3:**
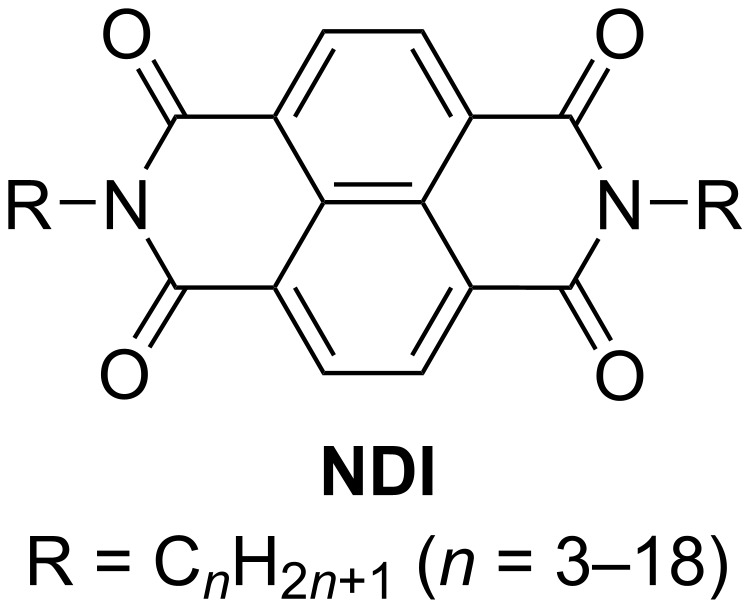
Chemical structure of the **NDI** derivatives [88].

**Figure 5:**
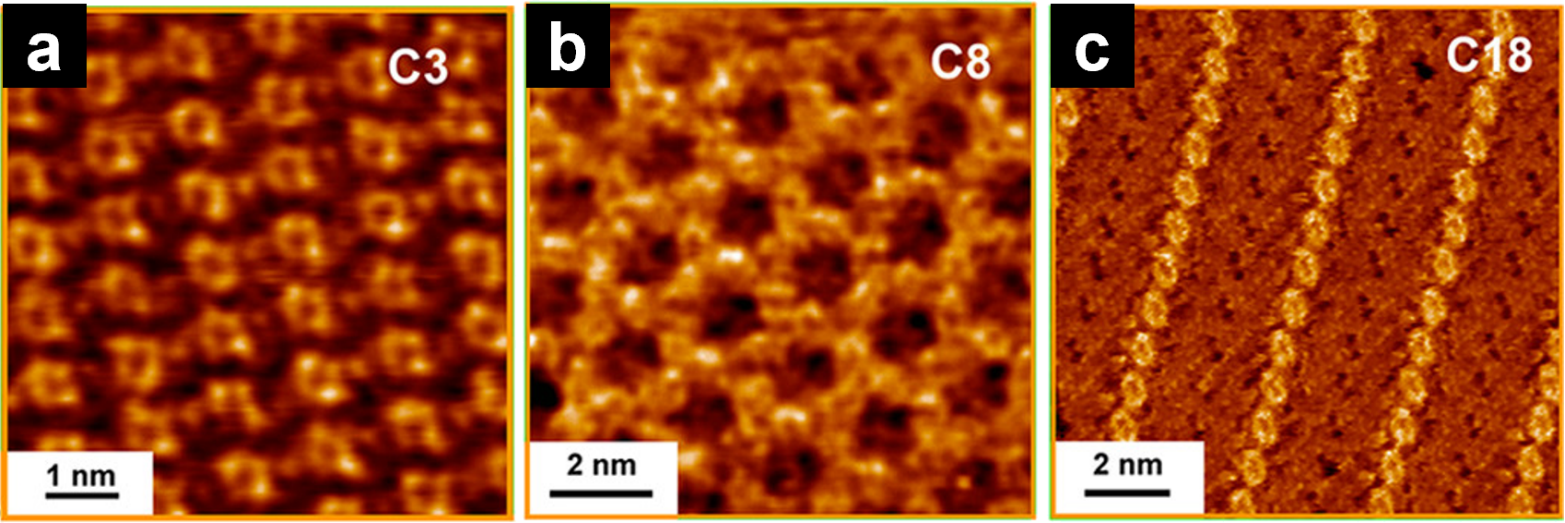
(a–c) Representative STM images of physisorbed monolayers of **NDI** formed at the HOPG/1–tetradecene interface: (a) lamellar; (b) honeycomb, and (c) lamellar structures. Figure 5 was adapted from [88], Copyright 2012 American Chemical Society. This content is not subject to CC BY 4.0.

The length of the alkyl chain affects the intermolecular distance between the functional units (often π-conjugated units) and the 2D molecular arrangements. For example, tetraalkoxydinaphthophenazines form columnar 2D structures [73]. The lattice constants and intercolumnar distances increased upon increase of the alkoxyl chain length (C4–C12). Similar changes in the intercolumnar distances caused by alkyl chain lengths have been reported for metalated bipyridine [74–78], isobutenyl ether compounds [79–84], and bissalicylaldiminatocopper(II) complexes [85], among others [86–87]. Regarding the alkyl-chain-length-dependent 2D molecular arrangements, self-assemblies of *N,N*′-bis(*n*-alkyl)naphthalenediimides (**NDI**) were systematically studied with the alkyl chain lengths ranging from C3 to C18 (Scheme 3) [88]. **NDI**s with short chains (C3 and C4, Figure 5a) and long chains (C13–C18, Figure 5c) showed lamellar structures, whereas those with medium length (C5–C12) formed honeycomb structures (Figure 5b), in which the alkyl chains were partially desorbed from the surface of HOPG. Desorption from the surface is unfavorable regarding enthalpy. However, the detached alkyl chain is mobile in the solution phase; thus, desorption from the surface is favorable concerning entropy. Therefore, the peculiar 2D structural change can be explained by the nonlinearity of the entropy term in the Gibbs free energy.

#### 3.2 Pore size

**Scheme 4:**
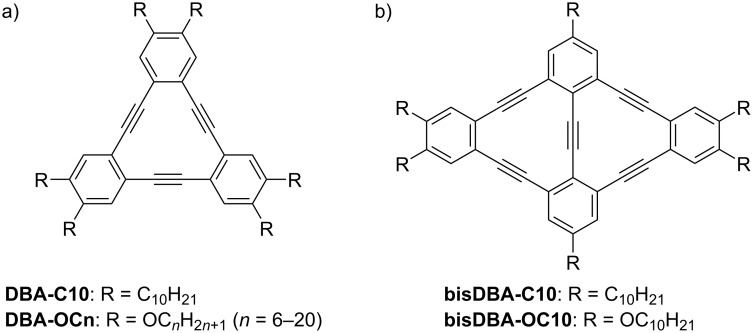
Chemical structures of (a) **DBA** and (b) **bisDBA** derivatives [91].

**Figure 6:**
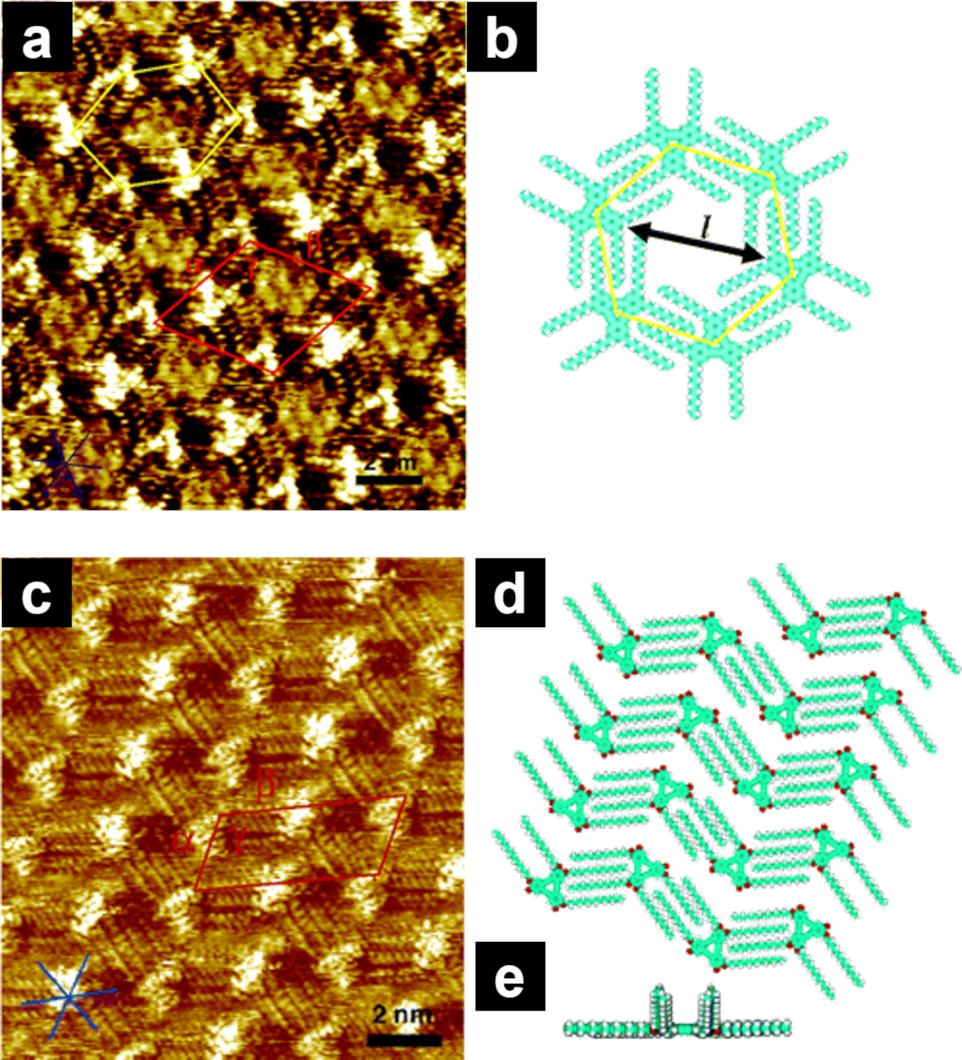
(a, c) STM images of physisorbed monolayers of **DBA-C10** and **DBA-OC14** formed at the HOPG/TCB interface, and (b, d, e) the corresponding molecular models. (a, b) Honeycomb structure of **DBA-C10**; (c–e) linear network of **DBA-OC14**. The symmetrical axes of HOPG are indicated in the lower left corner of the STM images. Figure 6 was adapted from [91], Copyright 2006 American Chemical Society. This content is not subject to CC BY 4.0.

The fabrication of nanoscale porous networks has attracted attention owing to their ability to accommodate guest molecules in the confined pores. Modification of the alkyl chain length facilitated the tuning of the pore size of the honeycomb structure. Dehydrobenzo[12]annulene (**DBA**, Scheme 4a) derivatives formed honeycomb structures in which the triangular **DBA** core was located at the vertices of the hexagon (Figure 6a,b) [89–90]. The honeycomb structure was highly stabilized by interdigitation of the four alkyl chains that followed the HOPG lattice directions. The pore size enlarged from 1.6 to 4.7 nm in accordance with the alkyl chain length ranging from C6 to C20, respectively [90]. In addition to the honeycomb structure, **DBA** formed wavy structures without pores [91], where two of the six alkoxy chains dangled into the solvent phase (Figure 6c–e). The formation of porous and non-porous networks depends on the alkyl chain length and the concentration of the alkoxy-substituted **DBA** derivatives [92]. Shorter alkoxy chains and lower concentrations are preferable for the formation of porous honeycomb structures, possibly because (i) **DBA**s with long alkoxy chains yield enough adsorption energy even when two of the four alkoxy chains do not participate in the adsorption and (ii) at low concentrations, a decreased number of building blocks can form a porous honeycomb structure with low density while increasing the adsorption energy per molecule (all alkyl chains are adsorbed). Adjusting the pore size by changing the alkyl chain length enables the accommodation of various guest molecules, resulting in multicomponent assemblies [68,93–98].

Note that **DBA-C10** and **DBA-OC10** with triangular core formed the same honeycomb structures, with only slightly different unit cell parameters. However, **bisDBA-C10** and **bisDBA-OC10** with lozenge-shaped core (Scheme 4b) exhibited Kagomé and other porous structures, respectively [91]. These results suggest that alkyl and alkoxy substitutions sometimes lead to different self-assembly behavior, which may be influenced by the structure of the molecular core.

#### 3.3 Opening and closing of pores

**Scheme 5:**
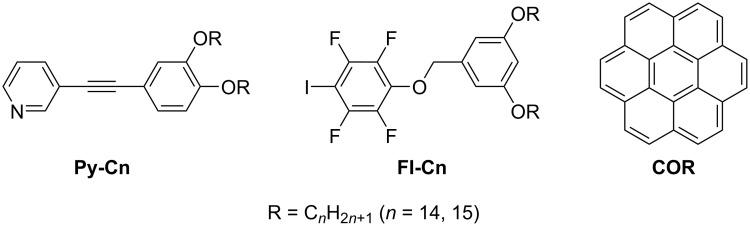
Chemical structures of pyridine-based (**Py-Cn**), tetrafluoroiodobenzene-based molecules (**FI-Cn**), and coronene (**COR**) [100].

**Figure 7:**
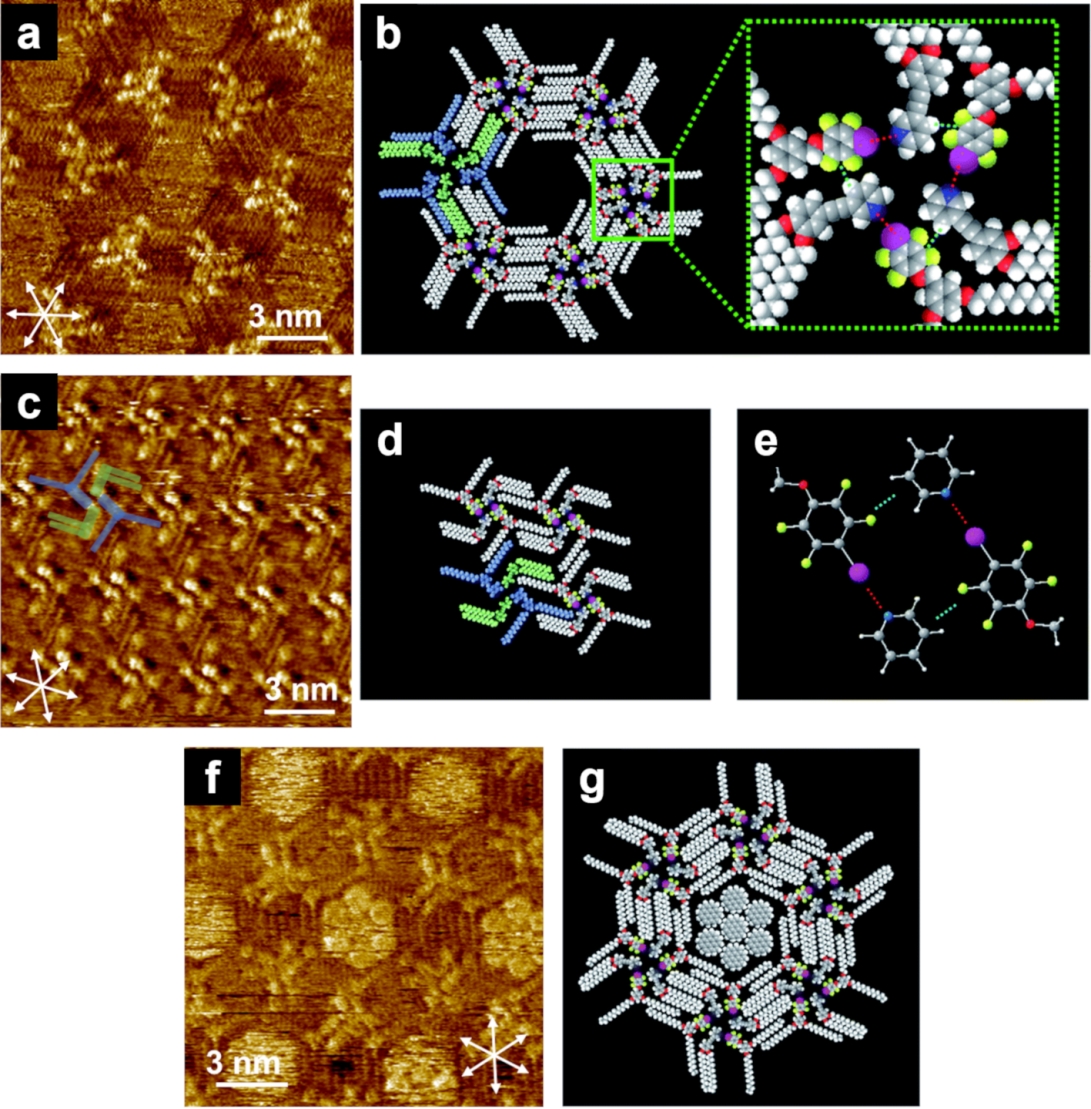
(a, c, f) STM images of blend systems in **Py-C15/FI-C15**, **Py-C14/FI-C14**, and **Py-C14/FI-C14/COR** at the HOPG/1-phenyloctane interface, and (b, d, e, f) molecular models. (a, b) Honeycomb structure of the **Py-C15/FI-C15** blend; (c–e) cross-shaped structure of the **Py-C14/FI-C14** blend; (f, g) honeycomb structure formed by the **Py-C14/FI-C14/COR** blend. The white arrows indicate the HOPG lattice directions. Figure 7 was adapted from [100], (“Dynamic host–guest behavior in halogen-bonded two-dimensional molecular networks investigated by scanning tunneling microscopy at the solid/liquid interface”, © 2020 Y. Kikkawa et al., published by The Royal Society of Chemistry, distributed under the terms of the Creative Commons Attribution-Non Commercial 3.0 Unported Licence, https://creativecommons.org/licenses/by-nc/3.0/). This content is not subject to CC BY 4.0.

Changes in the size and appearance of the porous structures have also been reported for halogen-bonded molecular networks comprising linear molecular building blocks (Scheme 5) [99–100]. The halogen bond donor and acceptor molecules individually exhibited different types of linear structures. Once these molecules were blended, cooperative I···N halogen bonding and Ar–F···H–Py interactions enabled the formation of triangular assemblies, which were organized into honeycomb arrays (Figure 7a,b). Such honeycomb structures were observed for molecules possessing alkyl chains, typically over C15. The shorter the alkyl chain length, the smaller the pore size. In contrast, component molecules with C14 chains displayed a cross-shaped morphology; nevertheless, the 2D structure was also stabilized by both I···N halogen bonding and Ar–F···H–Py interactions (Figure 7c–e). However, the addition of coronene as a guest allowed for a transformation of the 2D structure from cross-shaped to hexagonal, where a maximum of seven coronene molecules were close-packed and accommodated at the center of the hexagons (Figure 7f,g). Thus, the alkyl chain length affected the formation of porous or non-porous structures, whereas the incorporation of guest molecules changed the 2D assemblies, possibly due to the induced fit mechanism. Multicomponent assemblies owing to matching sizes between host networks and guest molecules have been also reported in **DBA** derivatives [68,96–98] and stilbene derivatives [101], among others [102–105].

### 4 Substitution positions and numbers

**Scheme 6:**
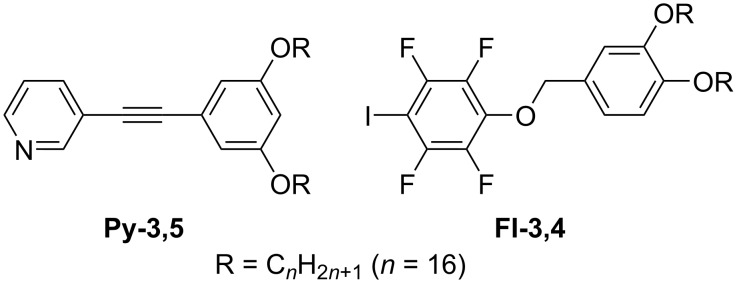
Chemical structures of **Py-3,5** and **FI-3,4** [109].

**Figure 8:**
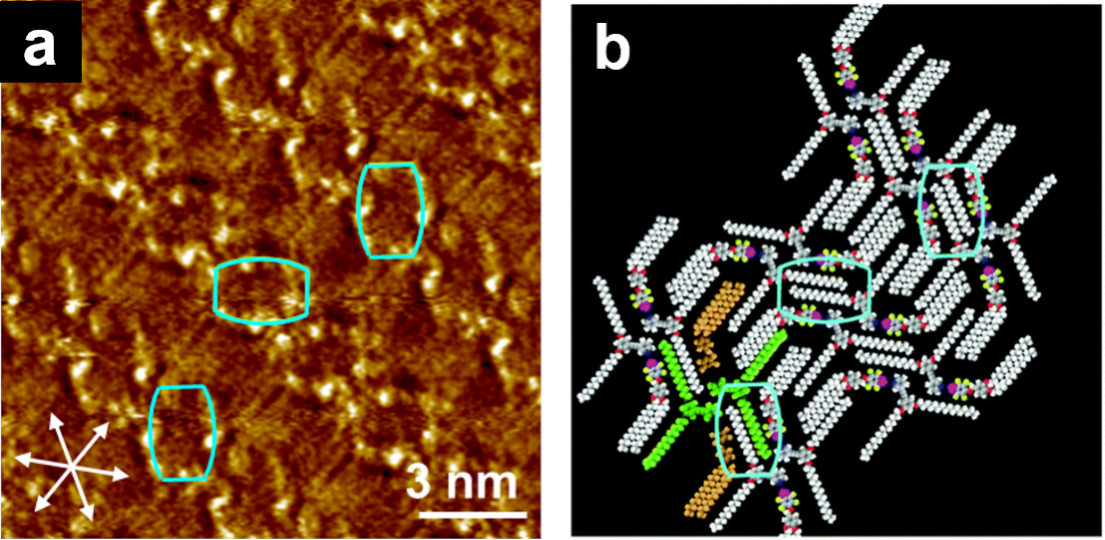
(a) STM image of the **Py-3,5/FI-3,4** blend at the HOPG/1-phenyloctane interface and (b) its molecular model. Rectangular assemblies highlighted in the cyan box are arranged in a zigzag fashion. The white arrows indicate the HOPG lattice directions. Figure 8 was adapted with permission of The Royal Society of Chemistry, from [109], (“Halogen bond-directed self-assembly in bicomponent blends at the solid/liquid interface: effect of the alkyl chain substitution position” by Y. Kikkawa et al., Physical Chemistry Chemical Physics, Vol. 24, Issue 28, © 2022); permission conveyed through Copyright Clearance Center, Inc. This content is not subject to CC BY 4.0.

Since the substitution positions of the alkyl chains alter the directions of the intermolecular dispersion interactions as well as the molecule–substrate interactions, the 2D structures can be modulated [106–108]. In the aforementioned halogen bonding system (section 3.3), the building blocks had different head groups, such as pyridine (**Py**) and tetrafluoro(iodo)benzene (**FI**), and they were substituted with alkyl chains at the 3,4- or 3,5-positions, respectively (hereafter, these molecules are denoted as **Py-3,4** and **FI-3,5**, Scheme 5). The combination of **Py-3,4** and **FI-3,5** enabled the formation of honeycomb structures (Figure 7a,b). However, the combination of **Py-3,5** and **FI-3,4** (Scheme 6) created a rectangular assembly arranged in a zigzag fashion (Figure 8a,b) [109]. The rectangular assembly involved only I···N halogen bonding, possibly because of the overlap of the alkyl chain units when **Py-3,5**/**FI-3,4** adopted a helical assembly in the **Py-3,4**/**FI-3,5** blend. To avoid wasting adsorption energy from the alkyl chains, the **Py-3,5/FI-3,4** blend adopted a rectangular assembly in which all the alkyl chains could adsorb on the HOPG surface.

**Scheme 7:**
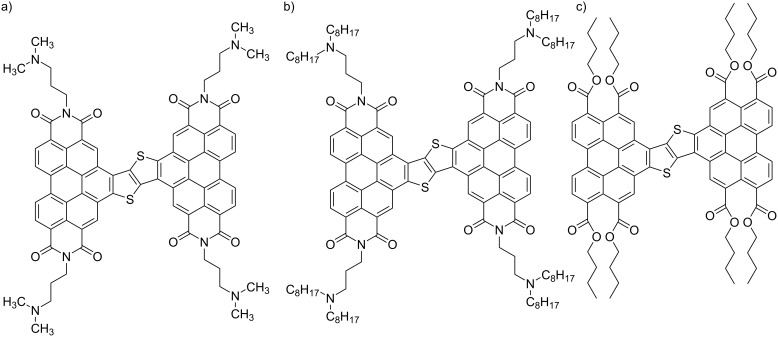
Chemical structures of perylene–bithiophene–perylene (**PBTP**) with (a) dimethylaminopropyl chains, (b) dioctylaminopropyl chains, and (c) butyl ester groups [110].

**Figure 9:**
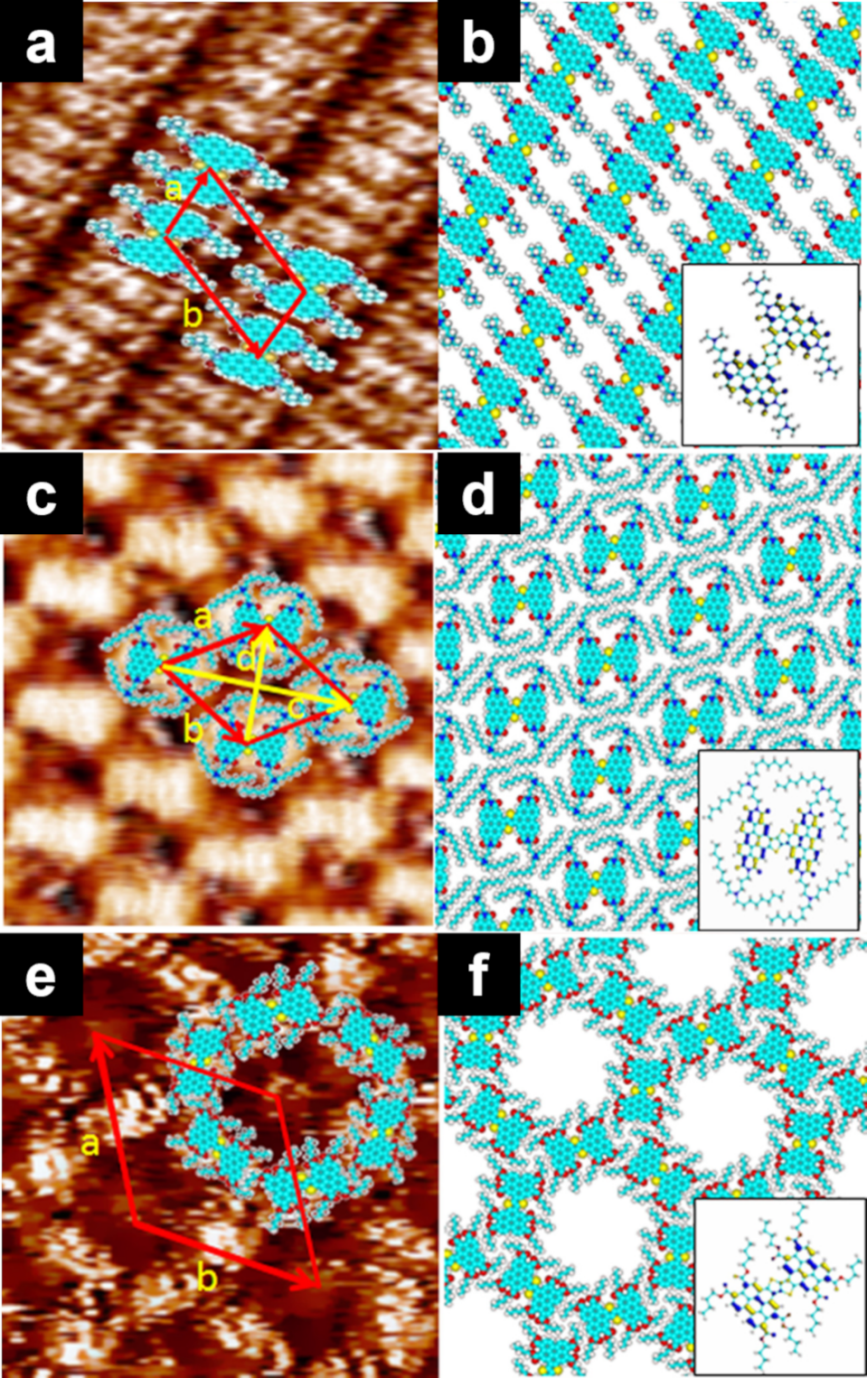
(a, c, e) STM images of the physisorbed monolayers at the HOPG/1-phenyloctane interface, and (b, d, f) molecular models. **PBTP** with (a, b) dimethylaminopropyl chains, (c, d) dioctylaminopropyl chains, and (e, f) butyl ester groups. Figure 9 was adapted from [110], Copyright 2016 American Chemical Society. This content is not subject to CC BY 4.0.

In addition to the substitution position, the number of the alkyl chain substitutions significantly affects molecular arrangement and orientation. In perylene–bithiophene–perylene derivatives (**PBTP**, Scheme 7), four N-positions of the imide groups were substituted with branched alkyl chains [110]. **PBTP** with four dimethylaminopropyl chains (Scheme 7a) was linearly arranged in a columnar structure (Figure 9a,b), whereas **PBTP** with four dioctylaminopropyl chains (Scheme 7b) exhibited different 2D structures in which the core unit of **PBTP** was surrounded by branched alkyl chains (Figure 9c,d). When **PBTP** was substituted with eight butyl ester groups at the 3, 4, 9, and 10 carbon positions of the two perylene motifs (Scheme 7c), porous honeycomb networks were created (Figure 9e,f). The flexibility of the alkyl chains and their steric hindrance enabled a match with the HOPG lattice and facilitated the rotation of the **PBTP** core into a hexagonal arrangement.

**Scheme 8:**
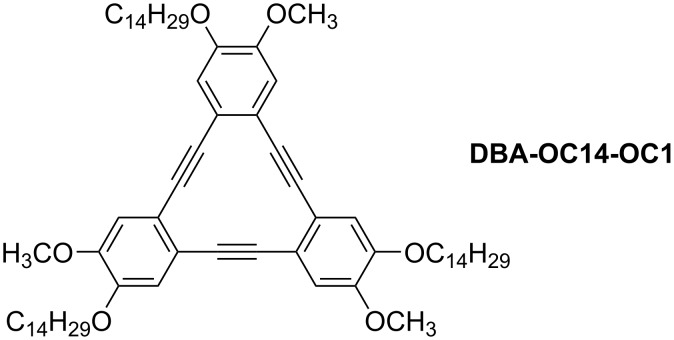
Chemical structure of **DBA-OC14-OC1** [111].

**Figure 10:**
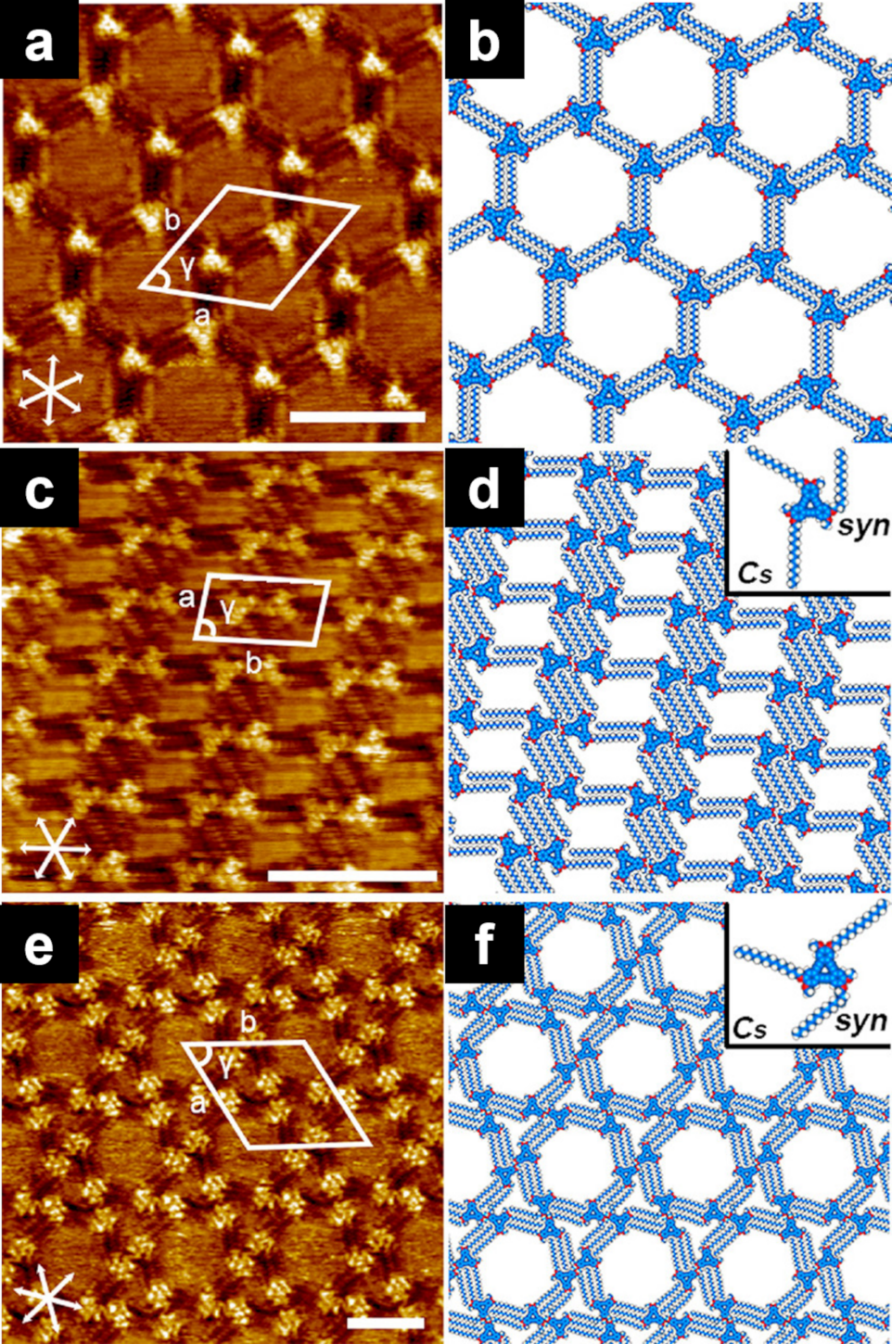
(a, c, e) STM images of the physisorbed monolayers at the HOPG/TCB interface and (b, d, f) their molecular models. (a, b) A honeycomb structure (3 × 10^−6^ M); (c, d) parallelogram structure (6 × 10^−5^ M); (e, f) hexagonal structure (7 × 10^−4^ M). Figure 10 was adapted from [111], Copyright 2019 American Chemical Society. This content is not subject to CC BY 4.0.

The number of alkyl chains controls the interaction modes, including interdigitation. A **DBA** derivative with six tetradecyloxy chains (**DBA-OC14**, Figure 6) exhibited honeycomb structures in which the alkyl chains were interdigitated [111]. The honeycomb structure was formed in a concentration range of 10^−6^–10^−4^ M, whereas a linear structure was formed at a high concentration of 7 × 10^−4^ M. In contrast, the **DBA** derivative modified with three tetradecyloxy chains and three methoxy groups at alternating positions (**DBA-OC14-OC1**, Scheme 8) was more sensitive to concentration alterations and showed various 2D structures such as porous honeycomb (3 × 10^−6^ M, Figure 10a,b), parallelogram (6 × 10^−5^ M, Figure 10c,d), and hexagonal patterns (7 × 10^−4^ M, Figure 10e,f) at the HOPG/TCB interface. By reducing the number of alkyl chains, the **DBA** derivative can adopt various conformations on the surface, forming polymorphic structures.

### 5 Odd–even effect

In addition to simple changes in the intermolecular distance, the number of carbon atoms in the alkyl chain sometimes enabled a drastic change in the 2D assemblies, owing to their odd–even nature. The odd–even effect of alkyl chains has been reported in 2D and 3D systems and is reflected in the periodic changes of characteristics such as morphology and physical properties [112–117]. The origin of the odd–even effect has been explained in terms of steric hindrance caused by the orientation of the terminal methyl group in the alkyl chain [43]. Therefore, even a small change in the number of CH_2_ units affects the molecular arrangement significantly, resulting in the diversification of 2D assemblies [76–77,81–84,118–138]. Some recent examples of the odd–even effect in 2D systems are presented below.

#### 5.1 Anthraquinone derivatives

**Scheme 9:**
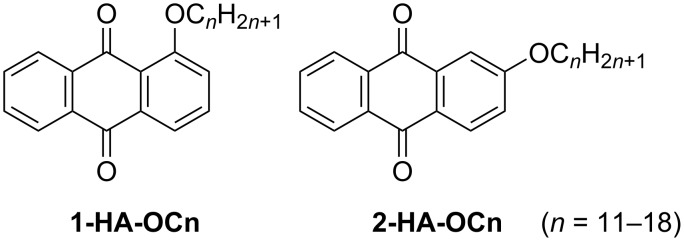
Chemical structures of **1-HA-OCn** and **2-HA-OCn** [129].

**Figure 11:**
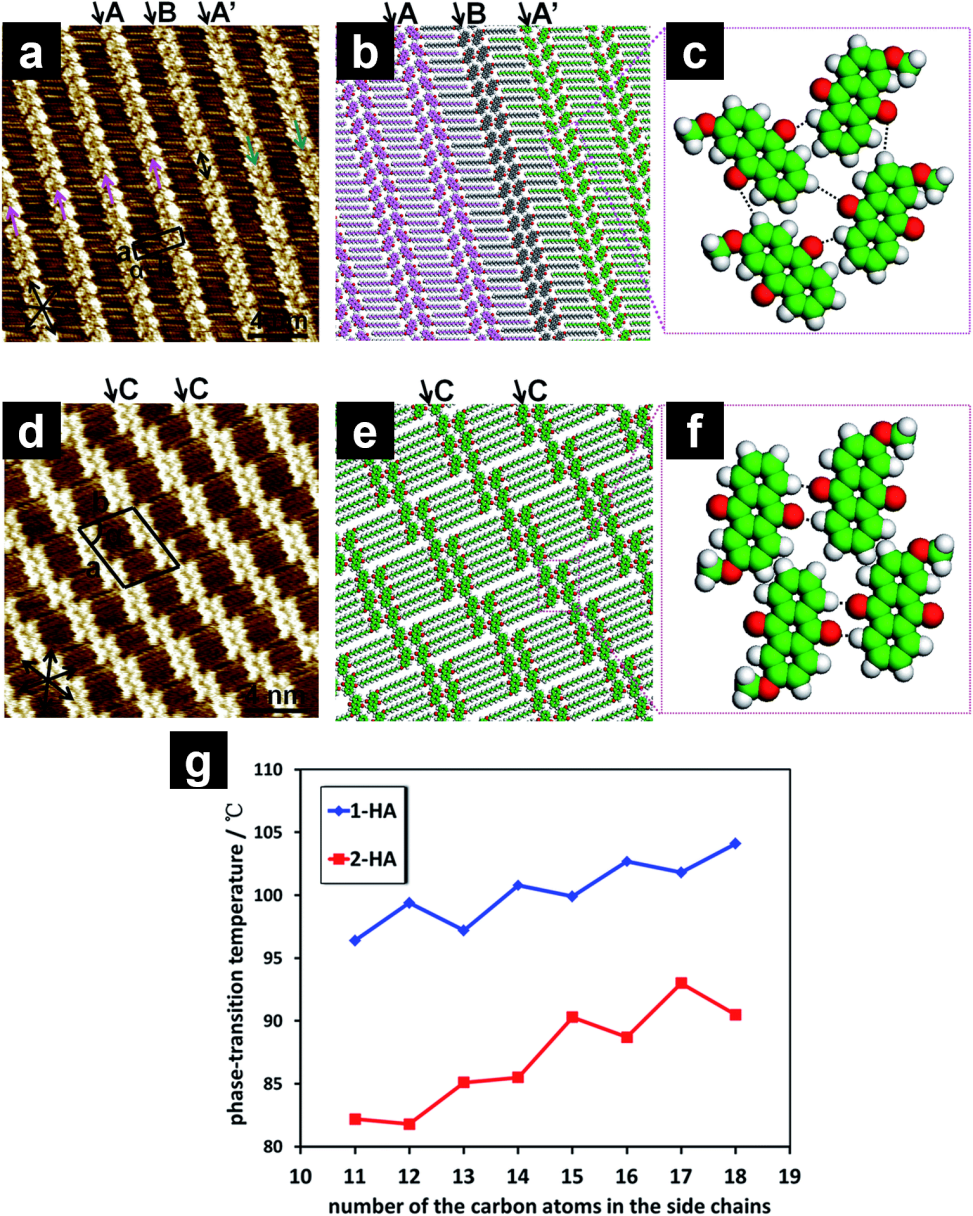
(a, d) STM images of the physisorbed monolayers at the HOPG/1-octanoic acid interface, (b, c, e, f) molecular models, and (g) the plots of phase transition temperature (melting point) for **1-HA-OCn** (blue) and **2-HA-OCn** (red) as a function of the number of carbon atoms in the alkyl chains. (a–c) Wheat-like structure formed by different orientations of anthraquinone pairs; (d–f) knot-like structure in which the clusters of the anthraquinone moieties shifted periodically. The arrows A–C indicate the different orientations of the anthraquinone cores (see text). Figure 11 was adapted with permission of The Royal Society of Chemistry, from [129], (“Side chain position, length and odd/even effects on the 2D self-assembly of mono-substituted anthraquinone derivatives at the liquid/solid interface” by Y. Hu et al., RSC Advances, Vol. 5, Issue 113, © 2015); permission conveyed through Copyright Clearance Center, Inc. This content is not subject to CC BY 4.0.

The 2D structures of anthraquinone derivatives (Scheme 9) substituted with a single alkoxy chain at the 1- (**1-HA-OCn**) and 2-position (**2-HA-OCn**) were studied by STM [129–130]. In both cases, weak O···H–C hydrogen bonding between adjacent anthraquinone moieties enabled pairing in a head-to-head manner. However, the orientation of the anthraquinone moieties depended on the (odd or even) number of carbon atoms in the interdigitated alkyl chains (C15 and C16), resulting in a periodic change in the 2D structures. Although both **1-HA-OC15** and **1-HA-OC16** displayed linear structures, the orientation of the anthraquinone moieties was different, forming parallel and V-shaped arrangements, respectively. A wheat-like structure was observed in the **2-HA-OC15** monolayer (Figure 11a–c), containing three different orientations of anthraquinone head groups (arrows A, A′, and B in Figure 11a,b). Lines A and A′ exhibited a V-shaped orientation of the head group pairs, while they were directed in opposite directions. In line B, the head groups exhibited parallel orientation. In contrast, **2-HA-OC16** exhibited a knot-like structure containing clusters of molecules (Figure 11d–f). In the cluster, two or three pairs of parallel oriented head groups (arrows C in Figure 11d,e) were arranged in a stepped manner with periodic shifts.

2D molecular arrangements are not completely the same as 3D crystals because of the presence of the substrate (HOPG) [137]. However, a correlation between the 2D structural modulation and the phase transition temperatures (melting point temperature *T*_m_) of **1-HA-OCn** and **2-HA-OCn** was revealed. The *T*_m_ values measured by differential scanning calorimetry (DSC) increased upon increase of the alkyl chain length exhibiting a zigzag fashion (Figure 11g) [129]. Such periodic changes in the 2D structure as well as *T*_m_ were also revealed for 2,6-bisalkoxy-substituted anthraquinone derivatives within the alkyl chain length range of C7–C18 [133]. STM, in conjunction with DSC studies, suggested that the balance between hydrogen bonding and dispersion interactions co-regulates *T*_m_. Other substitution effects, such as different substitution positions with two and three alkyl chains, were reported by the same group [139–140]. In addition, similar alkyl chain-related effects, including the odd–even effect, have been also reported for fluorenone derivatives [141–142].

#### 5.2 Isobutenyl ether compounds

**Scheme 10:**
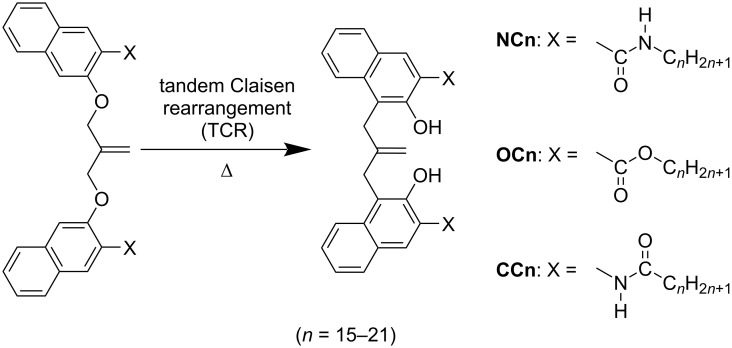
Chemical structures of isobutenyl ether compounds with differently linked alkyl chains before and after TCR [81–83,128,132].

**Figure 12:**
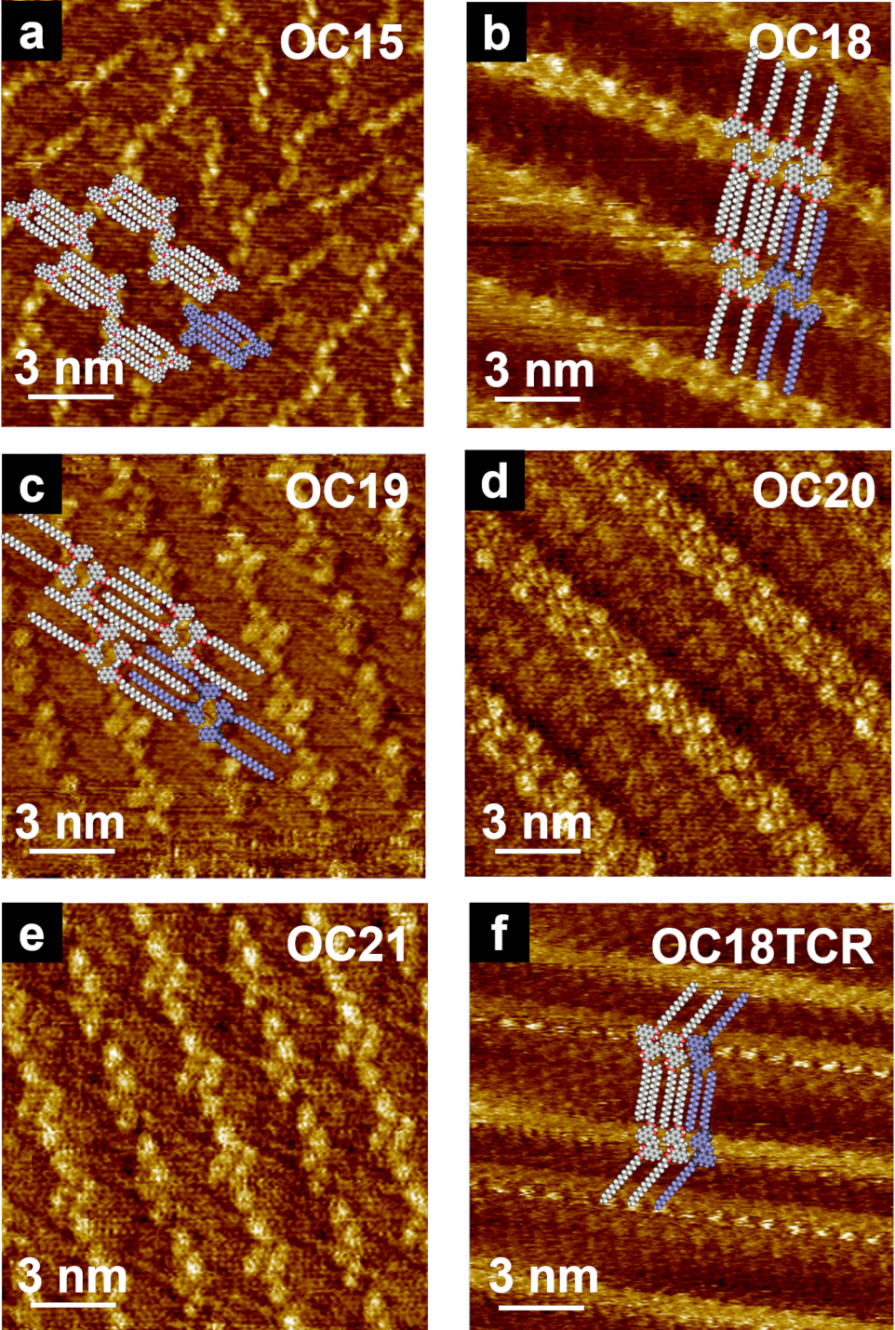
(a–f) STM images of the physisorbed monolayers at the HOPG/1-phenyloctane interface. The molecular models are superimposed on each STM image. (a–e) STM images of original **OC15** and **OC18**–**OC21** (f) STM image of **OC18** after TCR (**OC18TCR**). Figure 12 was adapted with permission of The Chemical Society of Japan from [83], (“Ester-Linked Alkyl Chain Effect on the 2D Structures of Isobutenyl Compounds: Scanning Tunneling Microscopic Study” by Y. Kikkawa et al., Bulletin of the Chemical Society of Japan 2015, Vol. 88, No. 6, 834–842. Figures 1A, D, E, G, H and 2D in [83] were combined to Figure 12 a–f, respectively. Copyright © 2015 The Chemical Society of Japan). This content is not subject to CC BY 4.0.

Isobutenyl ether compounds with either amide (**NCn**) [81–82] or ester-linked alkoxyl chains (**OCn**) exhibited 2D structural modulation owing to the odd–even effect when the alkyl chain length was in the range of C18–C21 (Scheme 10) [83]. **OC18** and **OC20** exhibited columnar structures in which their naphthalene units were arranged in a head-to-head fashion (Figure 12b,d). In contrast, in the monolayers of **OC19** and **OC21**, knot-like substructures were obliquely aligned along the columnar direction (Figure 12c,e). In the case of **OC19**, a tape-like structure was also observed. Thus, modulation of the 2D structure was observed for **OCn** (*n* ≥ 18) owing to the odd–even effect of the alkyl chains. Interestingly, **OCn** with shorter chains (*n* ≤ 17) exhibited a dumbbell-shaped structure without odd–even effect (Figure 12a). Isobutenyl ether compounds can be structurally converted by a thermal reaction of tandem Claisen rearrangement (TCR) [143]. TCR can transform an ether function into a hydroxy group forming new C–C bonds (Scheme 10). After TCR, intramolecular hydrogen bonds between the carbonyl and hydroxyl groups were introduced, and the distance between the two naphthalene units decreased, resulting in the naphthalene groups flipping in opposite directions due to their steric repulsion. The odd–even effect then disappeared and a converged linear structure was formed, regardless of the alkyl chain length (Figure 12f). Therefore, it was suggested that the molecular shape as well as the direction of the alkyl chain elongation affect the emergence of the odd–even effect.

#### 5.3 Blend systems

**Figure 13:**
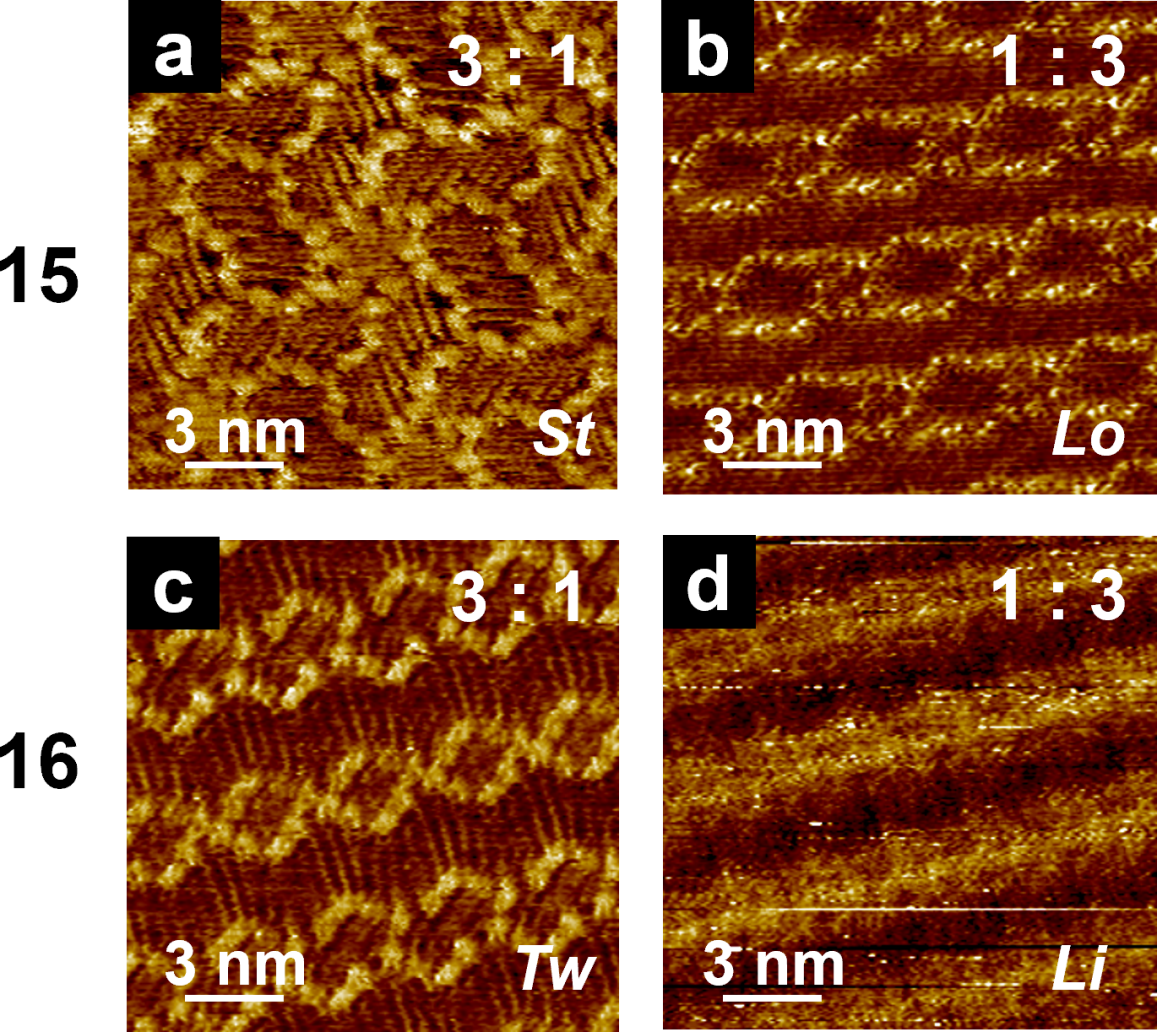
STM images of the **OCn** and **CCn** blends with different ratios: (a, b) *n* = 15 and (c, d) *n* =16. The blend ratio is shown in the right corner of each STM image in the order of **OCn**:**CCn**. The 2D structural features are indicated as star-like (*St*; a), lozenge-shaped (*Lo*; b), twist-like (*Tw*; c), and linear (*Li*; d) structures. Figure 13 was adapted with permission of The Royal Society of Chemistry, from [132], (“Odd–even effect in two dimensions induced by the bicomponent blends of isobutenyl compounds” by Y. Kikkawa et al., Physical Chemistry Chemical Physics, Vol. 19, Issue 21, © 2017); permission conveyed through Copyright Clearance Center, Inc. This content is not subject to CC BY 4.0.

Systems exist in which the individual components do not exhibit the odd–even effect, whereas the odd–even effect can be exhibited by a blend of them [128,132]. Isobutenyl ether compounds, in which the alkoxy chains (*n* = 15–18) were connected with ester (**OCn**) and carbamoyl linkages (**CCn**) were prepared (Scheme 10). Then, the 2D structure formation for individual molecules and bicomponent blend systems was studied by STM at the HOPG/1-phenyloctane interface. As stated in section 5.2, the naphthalene units of **OC18** were arranged in a columnar structure with a head-to-head orientation (Figure 12b), whereas those of **OCn** (*n* = 15–17) exhibited a dumbbell-shaped structure (Figure 12a). In the case of **CCn**, only columnar structures were formed. These results suggest that both **OCn** and **CCn** did not exhibit an odd–even effect within the range of C15–C18. Interestingly, bicomponent blends of **OCn** and **CCn** induced drastic 2D structural changes depending on the blend ratio and alkyl chain length, exhibiting star-like (blend ratio **OCn** > **CCn**, *n* = odd, Figure 13a), lozenge-shaped (**OCn** < **CCn**, *n* = odd, Figure 13b), twist-like (**OCn** > **CCn**, *n* = even, Figure 13c), and linear structures (**OCn** < **CCn**, *n* = even, Figure 13d). In situ addition of the blend partner enabled the alteration of the blend ratio, offering direct observation of the dynamic process of 2D structural changes in both ways, that is, from star to lozenge, from twist-like to linear structures, and vice versa. These structural differences were proposed to be introduced by homogeneous (**CCn**–**CCn**) and heterogeneous (**OCn**–**CCn**) dimers formed via interdigitation of alkyl chains. When the blend ratio was **OCn** > **CCn**, heterogeneous dimers of **OCn**–**CCn** constructed the 2D structures (star-like and twist-like structures). When **OCn** < **CCn**, the mixture of homogeneous (**CCn**–**CCn**) and heterogeneous (**OCn**–**CCn**) dimers formed lozenge and linear structures. Thus, the proportion of these interdigitated dimers determines the 2D structure of the blend system.

### 6 Chirality

**Figure 14:**
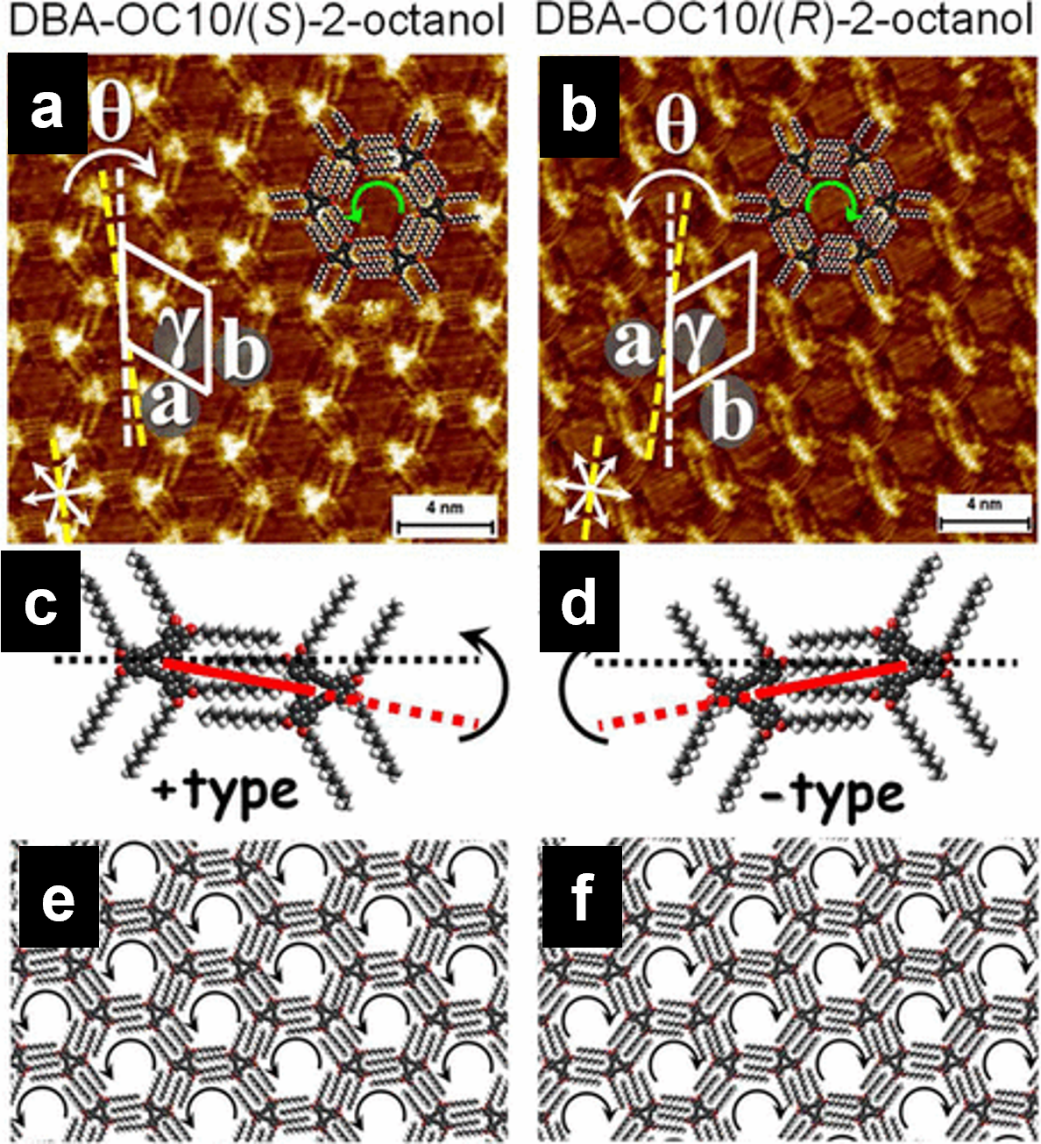
(a, b) STM images of **DBA-OC10** at the HOPG/(*S*)-2-octanol and HOPG/(*R*)-2-octanol interface, respectively. The yellow dashed lines indicate one of the HOPG lattice directions. θ is the angle between the HOPG axis and one of the unit cell vectors. (c, d) Interdigitation patterns of alkyl chains with different handedness in the molecular networks. (e, f) Molecular models for the self-assembled networks of **DBA-OC10** in (*S*)-2-octanol and (*R*)-2-octanol, respectively. Figure 14 was adapted from [144], Copyright 2012 American Chemical Society. This content is not subject to CC BY 4.0.

The chiral information of alkyl chain units can be transferred to supramolecular 2D assemblies, and the chirality of 2D structures composed of achiral molecular building blocks can be induced even upon the use of chiral solvents [144–147]. As stated in the above section 3.2, **DBA** derivatives formed hexagonal porous structures (Figure 6). Although the **DBA** molecule is achiral, clockwise (CW) and counter-clockwise (CCW) hexagons were observed, which were determined by the relative alignment of the interdigitation patterns of the alkyl chains (Figure 14c,d) [144]. **DBA-OC10** dissolved in (*S*)-2-octanol formed a CCW honeycomb structure (Figure 14a,e), whereas a CW structure was observed in the monolayer via self-assembly in (*R*)-2-octanol (Figure 14b,f). The induction of chirality was greater than 90% of the surface, and the CW and CCW domains were equally created when racemic 2-octanol was used. A pair of chiral solvents and **DBA** molecules formed upon interaction were proposed to act as precursors for nucleation on the surface, resulting in the induction of handedness in the 2D network. Chiral network formation was also demonstrated by installing chiral alkyl chains in the **DBA** [148–151] and 5-(benzyloxy)isophthalic acid cores [152].

### 7 Other effects

#### 7.1 Unsaturated alkyl chains

**Scheme 11:**
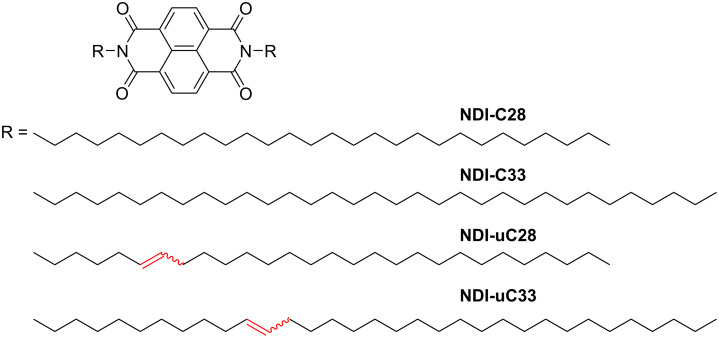
Chemical structures of **NDI-Cn** and **NDI-uCn** (*n* = 28 and 33) [153].

**Figure 15:**
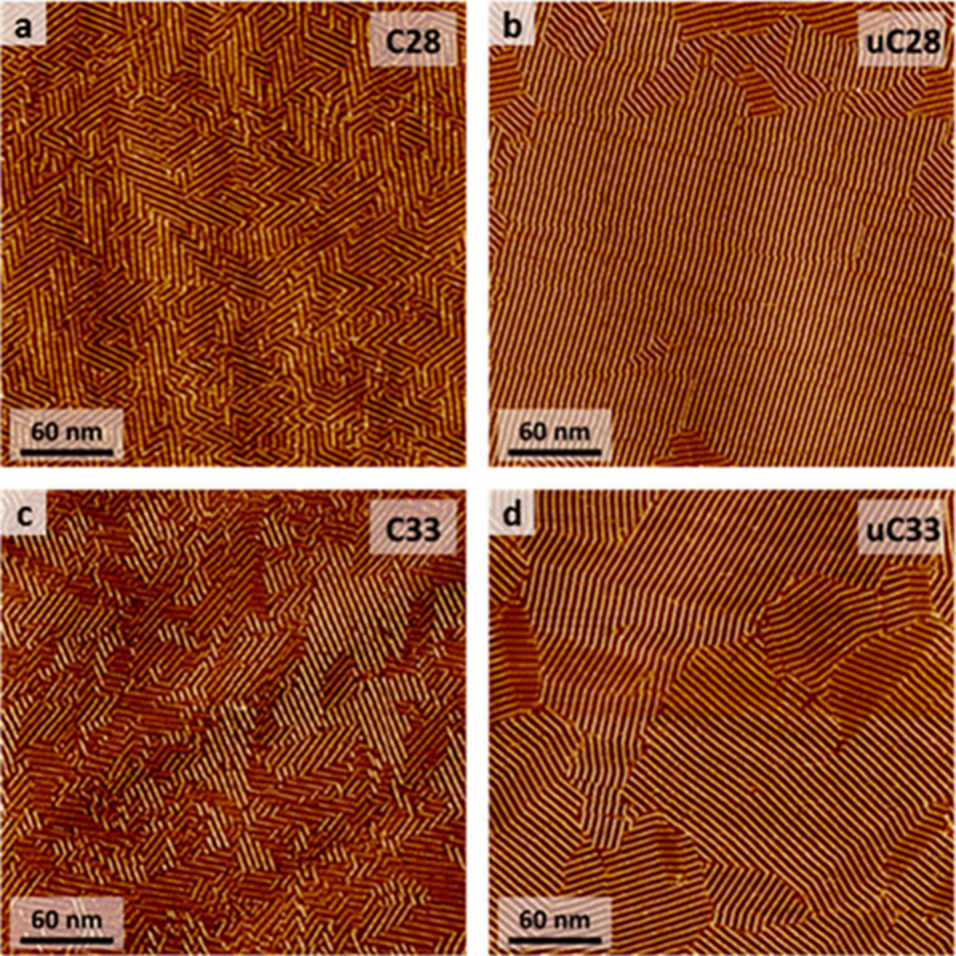
(a–d) Large-scale STM images (300 nm × 300 nm) of different **NDI** compounds at the HOPG/1-phenyloctane interface: (a) **NDI-C28**, (b) **NDI-uC28**, (c) **NDI-C33**, and (d) **NDI-uC33**. Figure 15 was reprinted from [153] (Berrocal, J. A. et al., Copyright 2020 American Chemical Society, distributed under the terms of the ACS AuthorChoice via Creative Commons Attribution Non-Commercial No Derivative Works 4.0 Usage Agreement, https://pubs.acs.org/page/policy/authorchoice_ccbyncnd_termsofuse.html). This content is not subject to CC BY 4.0.

2D ordering can be influenced by the absence or presence of double bonds in the aliphatic chain (i.e., saturated or unsaturated alkyl chains) [153]. Naphthalenediimide derivatives substituted with saturated alkyl chains (**NDI-Cn**, with either 28 or 33 carbon atoms in the chain, Scheme 11) formed columnar structures containing both interdigitated and non-interdigitated alkyl chain arrangements. However, **NDI** possessing unsaturated alkyl chains (**NDI-uCn**) favored interdigitated arrangements, resulting in the formation of well-organized 2D structures. Therefore, **NDI-uCn** formed larger domains with fewer defects than **NDI-Cn** (Figure 15a–d). Such large-area ordering due to the interdigitated structure was attributed to the larger dispersion interactions of the unsaturated chains compared to that of the fully saturated ones.

The stabilizing effect of unsaturated alkyl chains has been also reported for **DBA** derivatives [154]. The introduction of a diacetylene unit in the alkyl chain enabled the formation of honeycomb structures with larger pore sizes than those of their counterparts with *n*-alkyl chains.

**Figure 16:**
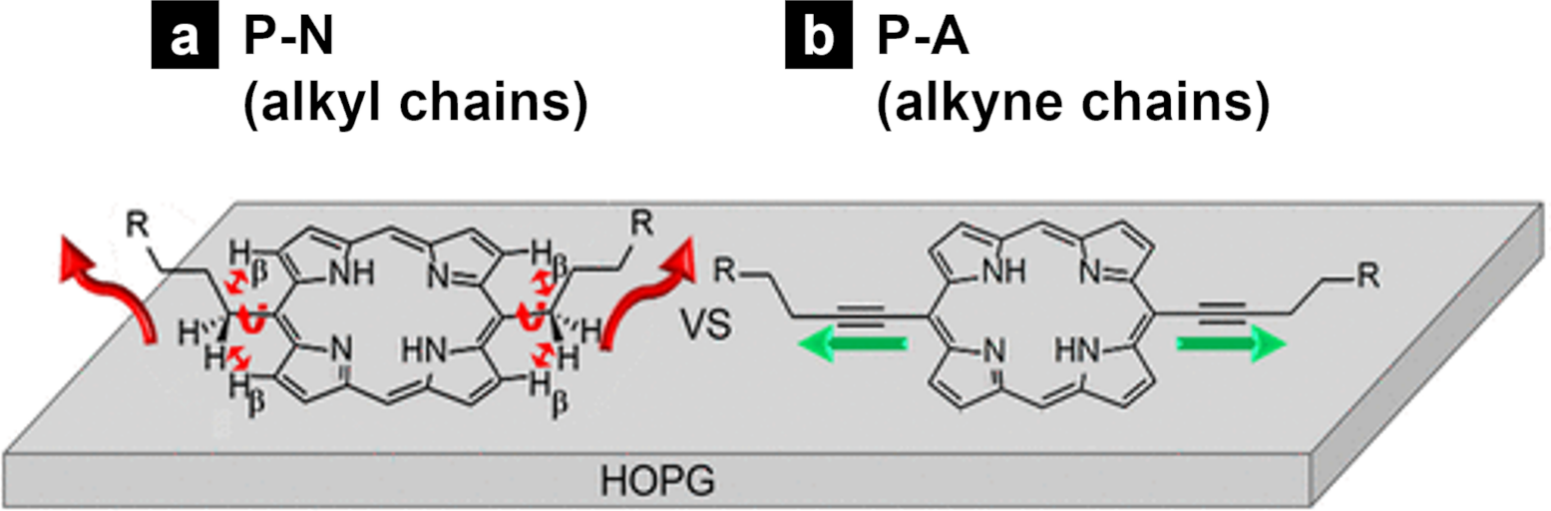
Schematic representation of porphyrin substituted with (a) normal alkyl (**P-N**) and (b) alkynyl chains (**P-A**). The alkyl chains of **P-N** tend to extend in the solvent phase, whereas alkyne chains of **P-A** lead to a flat orientation. Figure 16 was adapted from [155], Copyright 2020 American Chemical Society. This content is not subject to CC BY 4.0.

Alkynyl chains (alkyl chains with triple bonds) allow for a change in the tilt angle between the core unit and aliphatic chains. When the meso positions of porphyrins were substituted with normal saturated alkyl chains (**P-N**), the porphyrin plane and the alkyl chain units exhibited torsional strain, resulting in a tilt angle between the porphyrin core and the direction of the alkyl chain extension (Figure 16a). However, the meso-alkyne-substituted porphyrin (**P-A**) could be co-planar (Figure 16b). Therefore, **P-A** could form fully covered monolayers, even at a lower concentrations than **P-N**. This result suggests that alkynyl linkers contribute more to the monolayer stabilization of porphyrin derivatives than normal alkyl chains, because of the flat orientation of the alkynyl chains [155].

#### 7.2 Fluoroalkyl chains

Fluoroalkyl chain units have been rarely used for self-assembly at the HOPG/solvent interface because molecules substituted with only fluoroalkyl chains cannot form a stable monolayer, thus disabling STM observation [58]. Dispersion-corrected DFT calculations offered quantitative information on the interactions between *n*-perfluoroalkanes and circumcoronene (HOPG model) [47]. Compared to the *n*-alkyl chains, several disadvantages of the *n*-perfluoroalkyl chains can be suggested for the assembly on the graphite surface: (1) The adsorption energies of *n*-perfluoroalkanes are significantly smaller than those of *n*-alkanes. (2) A lattice mismatch between *n*-perfluoroalkane and circumcoronene occurs in the close-packed structure. (3) The change in the adsorption energy by the rotation of adsorbed *n*-perfluoroalkanes is smaller than that of the corresponding *n*-alkanes.

**Scheme 12:**
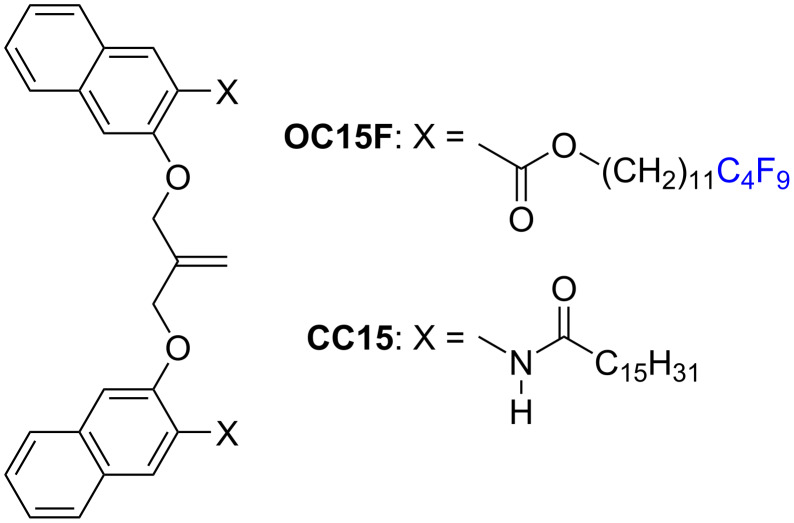
Chemical structures of **OC15F** and **CC15** [132].

**Figure 17:**
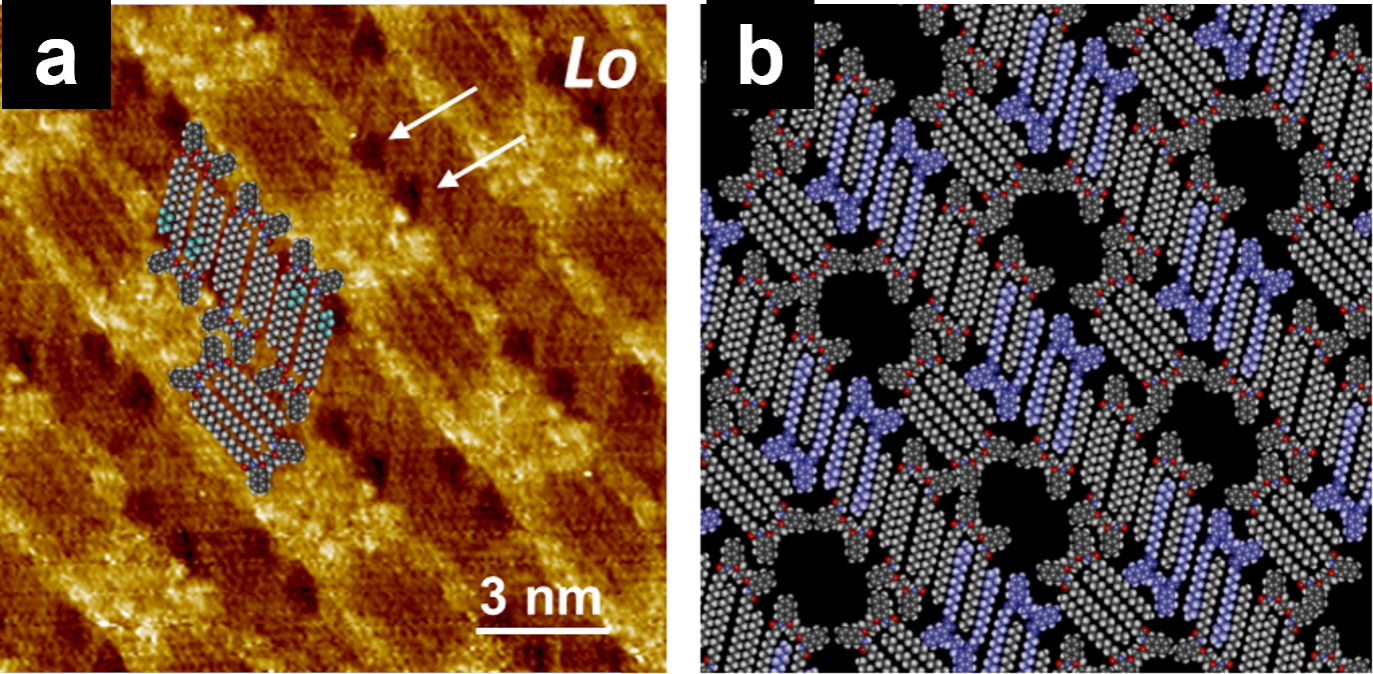
(a) STM image and (b) molecular model of the self-assembled structure of the **OC15F/CC15** blend at the HOPG/1-phenyloctane interface. Arrows in (a) indicate the typical area showing a darker contrast due to the semi-fluoroalkyl chains. In (b), **OC15F** molecules are highlighted in blue. The formation of homogeneous (**CC15**–**CC15**) and heterogeneous (**OC15F**–**CC15**) dimers is obvious. Figure 17 was adapted with permission of The Royal Society of Chemistry, from [132], (“Odd–even effect in two dimensions induced by the bicomponent blends of isobutenyl compounds” by Y. Kikkawa et al., Physical Chemistry Chemical Physics, Vol. 19, Issue 21, © 2017); permission conveyed through Copyright Clearance Center, Inc. This content is not subject to CC BY 4.0.

Fluoroalkyl chains appear as a darker contrast in STM images compared to *n*-alkyl chains. They have therefore been used as chemical markers to identify the location of molecular species [156–160]. For example, in a blended system of isobutenyl ether compounds (see section 5.3), **OCn** with a semi-fluoroalkyl chain (**OC15F**) was mixed with **CC15** (Scheme 12) [132]. Although the blend ratio of **OCnF**:**CCn** was 3:1, the structure was lozenge-shaped (Figure 17a,b), which should be formed when **OCn** was blended with **CCn** at a blend ratio of **OCn**:**CCn** = 1:3. This phenomenon could be explained by the relatively weak adsorption interactions of the semi-fluoroalkyl chains onto the HOPG surface compared to those of normal alkyl chains. Owing to the dark contrast in the STM image, the existence and location of homogeneous (**CC15**–**CC15**) and heterogeneous (**OC15F**–**CC15**) dimers were clearly ascertained. This identification by STM and DFT calculations enabled the authors to propose a mechanism for a 2D structural diversification of the bicomponent blend.

#### 7.3 Alkyl chains of solvent molecules

**Figure 18:**
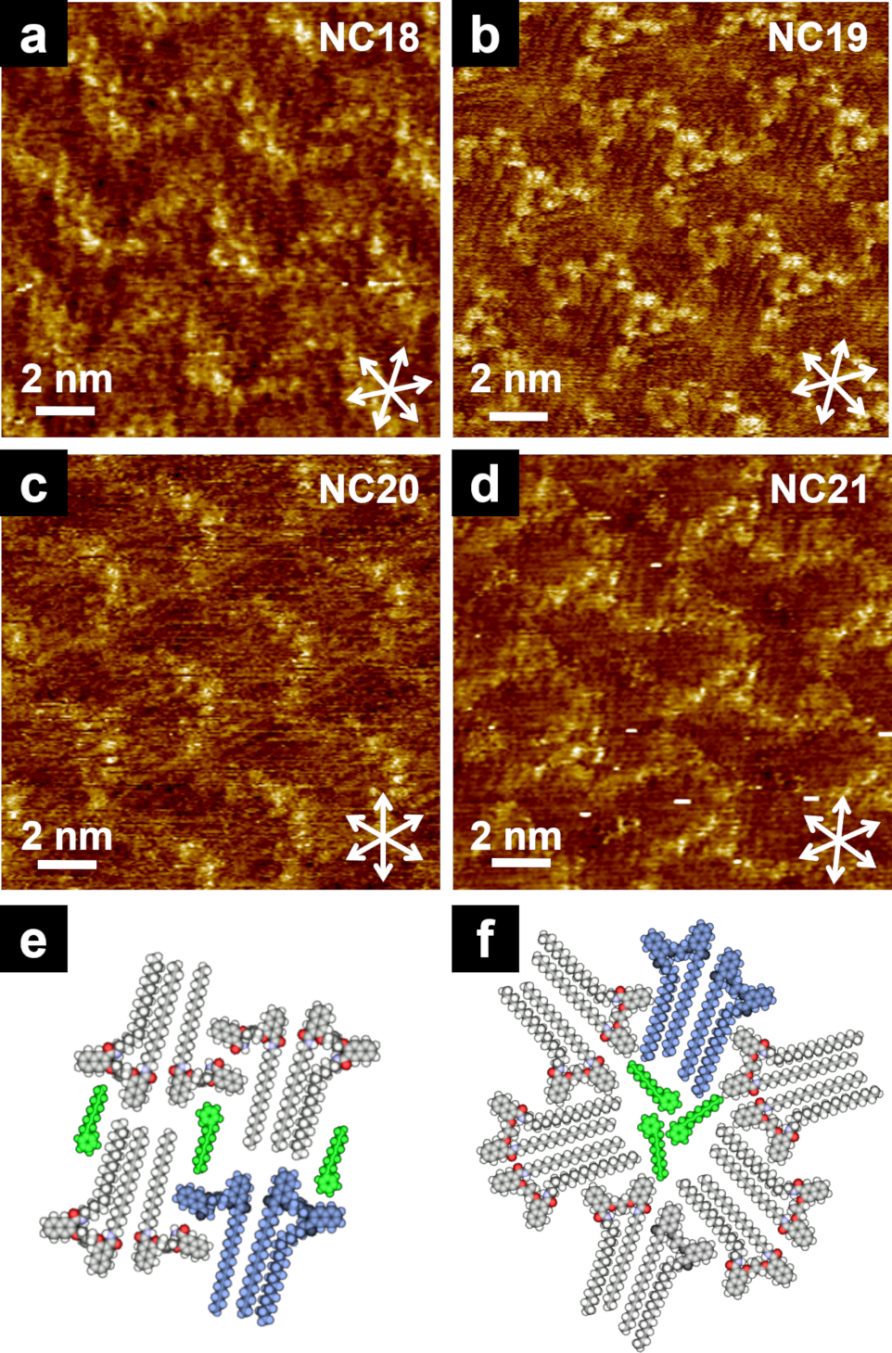
(a–d) STM images of **NCn** (*n* = 18–21) physisorbed at the HOPG/1-phenylnonane interface. Molecular models of (e) wavy and (f) tripod structures. One pair of **NCn** and the co-adsorbed 1-phenylnonane molecules are colored in blue and green, respectively. Figure 18 was adapted with permission of The Royal Society of Chemistry, from [82], (“Effects of alkyl chain length, solvent and tandem Claisen rearrangement on two-dimensional structures of noncyclic isobutenyl compounds: scanning tunnelling microscopic study” by Y. Kikkawa et al., Organic & Biomolecular Chemistry, Vol. 10, Issue 40, © 2012); permission conveyed through Copyright Clearance Center, Inc. This content is not subject to CC BY 4.0.

Co-adsorption of the solvent can occur due to the similar sizes of the void space and solvent molecules as well as due to the dispersion interactions between the alkyl chains of the building blocks and the solvent molecules. For example, 2D structures of isobutenyl ether compounds with amide-linked alkyl chains (**NCn**: *n* = 18–21, Scheme 9) alternately changed due to the “weak” odd–even effect of the alkyl chains at the HOPG/1-phenyloctane interface. **NC18** formed a wavy structure, whereas **NC19** and **NC21** displayed C3 symmetric tripod structures [82]. In **NC20**, both wavy and tripod structures appeared in separate domains. However, at the HOPG/1-phenylnonane (C9) interface (instead of 1-phenyloctane (C8)), **NC20** only exhibited a wavy structure. This is possibly due to the alkyl chain length of the solvent, enabling the convergence of the 2D structure to a wavy structure, resulting in the emergence of a “complete” odd–even effect. **NC18** and **NC20** formed a pair, while one of the four alkyl chains dangled into the solvent phase, whereas the space in the wavy structure was filled with the co-adsorbed solvent molecules (Figure 18a,c). In the tripod structure, pairs of **NC19** and **NC21** were arranged along the threefold symmetric directions of HOPG. All alkyl chains were adsorbed on the HOPG surface, and three solvent molecules were incorporated into the central vacant space (Figure 18b,d). These results suggest that the different chain lengths of the co-adsorbed solvent affect the intermolecular interactions of the alkyl chain terminals, resulting in structural divergence/convergence, especially in **NC20**.

## Conclusion

In this review, we introduce and summarize the significant effects of alkyl chains on 2D self-assemblies, demonstrated in various STM studies at the solid/liquid interface. The major roles of alkyl chains can be summarized as follows: (i) The alkyl chains assist adsorption onto the HOPG surface with epitaxy, enabling the formation of an oriented physisorbed monolayer. Long alkyl chains can have a strong stabilization energy for adsorption, comparable to other strong supramolecular interactions such as hydrogen bonds. (ii) The length of the alkyl chains determines the intermolecular distance between the core unit as well as the size of the porous networks. (iii) Odd or even numbers of carbon atoms in the alkyl chain result in periodic changes in the 2D structures, allowing for the diversification of the 2D patterns (odd–even effect). (iv) Chiral information of alkyl chain units can induce 2D chirality of molecular networks. (v) Molecules with unsaturated alkyl chains enable the formation of more ordered 2D structures than those with saturated alkyl chains. (vi) Fluoroalkyl chains can serve as chemical markers to identify their location and orientation, although the adsorption ability of fluoroalkyl chains is lower than that of normal alkyl chains. Of course, these alkyl chain effects cannot be completely isolated and discriminated from other intermolecular interactions. However, as reported in many publications, alkyl chains have a great influence on the formation of 2D nanoarchitectures. Recent advances in 2D self-assembly include “on-surface synthesis,” “in situ reactions” such as metal coordination, “correlation of the 2D and 3D structures (crystal structures)” for revealing the origins of physical properties, and “covalent functionalization” using 2D structures as templates [161–171]. In these systems, the molecular building blocks mostly comprise alkyl chains, and various phenomena, including the interaction of alkyl chains, continue to be revealed. Therefore, understanding the effects of alkyl chains and combining them with other interactions is an important strategy in the design and control of 2D nanoarchitectures. We hope that this review can evoke the unprecedented idea of nano-architecture design for the application regarding nanodevices and nanopatterning using functional organic molecules, based on the concept of nanoarchitectonics.
